# Effectiveness of low-dose amitriptyline and mirtazapine in patients with insomnia disorder and sleep maintenance problems: a randomised, double-blind, placebo-controlled trial in general practice (DREAMING)

**DOI:** 10.3399/BJGP.2024.0173

**Published:** 2025-06-17

**Authors:** Mette H Bakker, Jacqueline G Hugtenburg, Pierre M Bet, Jos WR Twisk, Henriëtte E van der Horst, Pauline Slottje

**Affiliations:** Department of General Practice, Amsterdam University Medical Center (UMC); Amsterdam Public Health Research Institute, Quality of Care, Amsterdam; Department of Clinical Pharmacology and Pharmacy, Amsterdam UMC; Amsterdam Public Health Research Institute, Quality of Care, Amsterdam; Department of Clinical Pharmacology and Pharmacy, Amsterdam UMC, Amsterdam; Department of Epidemiology and Data Science, Amsterdam UMC, Amsterdam; Department of General Practice, Amsterdam University Medical Center (UMC); Amsterdam Public Health Research Institute, Quality of Care, Amsterdam; Department of General Practice, Amsterdam University Medical Center (UMC); Amsterdam Public Health Research Institute, Quality of Care, Amsterdam

**Keywords:** amitriptyline, insomnia, mirtazapine, primary health care

## Abstract

**Background:**

Low-dose amitriptyline and mirtazapine are widely prescribed off-label for insomnia disorder. However, evidence of their effectiveness from placebo-controlled studies is lacking.

**Aim:**

To assess the effectiveness of low-dose mirtazapine and amitriptyline in patients with insomnia disorder.

**Design and setting:**

Pragmatic, double-blind, randomised, placebo-controlled trial undertaken in general practices in the Amsterdam region, the Netherlands.

**Method:**

Patients (aged 18–85 years) with insomnia disorder and sleep maintenance problems for whom non-pharmacological treatment was insufficient were randomised to mirtazapine (7.5–15 mg/day), amitriptyline (10–20 mg/day), or placebo for 16 weeks (optional double-dose regime in week 2–14). Insomnia Severity Index (ISI) scores (range 0–28) were assessed at baseline and again at 6, 12, 20, and 52 weeks. The primary outcome was an ISI total score at 6 weeks that was clinically relevant and signified either ‘improvement’ (>7 points lower than baseline) or ‘recovery’ (total score ≤10 points).

**Results:**

In total, 80 participants were included. At 6 weeks, in the intention-to-treat analyses, mirtazapine and amitriptyline each led to statistically significantly lower ISI scores when compared with placebo: mirtazapine mean difference = −6.0 points (95% confidence interval [CI] = −9.0 to −3.0), amitriptyline mean difference = −3.4 points (95% CI = −6.3 to −0.4). At 6 weeks mirtazapine resulted in statistically significantly higher improvement and recovery rates (52% and 56%, respectively) compared with placebo (both 14%), whereas amitriptyline (with rates of 40% and 36%, respectively) did not. From 12 weeks onwards no statistically significant differences in ISI scores were observed.

**Conclusion:**

Compared with placebo, low-dose mirtazapine provided a statistically significant and clinically relevant reduction of insomnia severity at 6 weeks, but not at later time points. Low-dose amitriptyline resulted in a statistically significant reduction at 6 weeks, but this was not clinically relevant. The results do not support the prescription of low-dose amitriptyline and mirtazapine for several months in patients with insomnia disorder in general practice. Based on the results, GPs may consider prescribing off-label low-dose mirtazapine for a period of about 6 weeks in case non-pharmacological treatment is insufficient.

## Introduction

Insomnia disorder is a common sleep disorder with a chronic course that poses a considerable burden on patients and society.^1^,^2^ To meet the diagnostic criteria, there must be a predominant complaint of dissatisfaction with sleep quantity or quality for ≥3 days a week for ≥3 months, resulting in significant daytime impairment despite sufficient opportunity to sleep.^3^

Treating insomnia disorder is challenging, as the first-choice treatment — namely, cognitive behavioural therapy for insomnia (CBT-I) — is not suitable or sufficiently effective for all patients.^4^–^9^ Licensed benzodiazepine receptor agonist sleep medication has an established short-term efficacy, but its use may result in serious adverse effects and is only recommended for periods of <4 weeks.^4^,^10^–^12^ Low doses of the antidepressants amitriptyline and mirtazapine are widely prescribed as an alternative treatment when there is a need for sleep medication that can be used for several months.^13^–^17^ Both generic drugs are used in higher dosages to treat depression and anxiety in primary and secondary care. Clinical observations, the pharmacological mechanism of action, and a few non-controlled studies have suggested that these drugs may enhance sleep maintenance;^18^–^20^ however, they are not licensed for this indication and placebo-controlled studies to either support or discourage this off-label prescription practice are lacking.^14^,^21^ As such, a randomised, double-blind, placebo-controlled trial was conducted in patients with insomnia disorder in general practice to assess the effectiveness of low-dose amitriptyline and mirtazapine during a 16-week treatment period, with up to 52 weeks’ follow-up.

**How this fits in**

The antidepressants amitriptyline and mirtazapine are widely prescribed in low doses for several months to patients with insomnia disorder, although they are not licensed for this indication. Placebo-controlled studies on the short- and long-term effectiveness of these drugs in patients with insomnia disorder are lacking. This trial involving patients with insomnia disorder in general practice suggested that low-dose mirtazapine (7.5–15 mg/day) provides a statistically significant and clinically relevant reduction in insomnia severity up to 6 weeks, but offers no benefit over placebo from 12 weeks onwards. When compared with placebo, low-dose amitriptyline resulted in a statistically significant, but not clinically relevant, reduction in insomnia severity at 6 weeks only.

## Method

### Design

The Drug REdiscovery: low-dose Amitriptyline and Mirtazapine for INsomnia disorder in General practice (DREAMING) study was a pragmatic, investigator-initiated, individually randomised, double-blind, placebo-controlled, multicentre, phase-III trial in general practice with three parallel treatment groups (ratio 1:1:1): mirtazapine, amitriptyline, and placebo. It was coordinated by the Department of General Practice at Amsterdam University Medical Center in the Netherlands, in collaboration with 64 general practices in the Amsterdam region. A detailed study protocol explaining the pragmatic approach has been published elsewhere.^22^ The trial was conducted in accordance with the International Conference for Harmonisation Good Clinical Practice regulations (ICH-GCP).^23^ The authors followed the CONsolidated Standards Of Reporting Trials (CONSORT) guidelines for randomised trials^24^ and the extension on pragmatic trials.^25^

### Participating practices

Recruitment of participating general practices took place via existing contacts with care groups in the Amsterdam region. More participating practices took part than initially planned (64 instead of 50 sites) and the patient inclusion period was prolonged (30 months instead of 18 months).

### Participants

Participants were recruited on consultation by their own GP. The target group consisted of patients who:

- were aged 18–85 years;
- had been diagnosed with insomnia disorder in general practice;
- were experiencing difficulty maintaining sleep or early-morning awakening problems; and
- consulted their GP with a request for sleep medication and for whom non-pharmacological treatment was deemed insufficient by the patient and the GP.

The eligibility and consent procedures and selection criteria are detailed in Supplementary Box S1.

### Study medication

The study medication comprised identically appearing round, white, film-coated tablets that contained amitriptyline 10 mg, mirtazapine 7.5 mg, or the placebo containing denatonium benzoate (for a slightly bitter taste).^22^

### Intervention and procedures

The intervention consisted of a 16-week daily intake of either one or two tablets of mirtazapine 7.5 mg, amitriptyline 10 mg, or placebo. Patients were instructed to start with one tablet per night, taken 2 hours to 30 minutes before bedtime. In a pre-planned follow-up consultation that took place in week 2, 3, or 4, the GP and participant could choose to double the dose per night, up to week 14; all participants were instructed to use a single dose in the last 2 weeks. Participants on a double-dose regime were allowed to return to a daily single-dose regime earlier after discussion with their GP.

At the preplanned consultation that took place in week 13 or 14, the GP informed the participant about potential short-term rebound effects after stopping the medication. After stopping, participants were instructed to return the remaining study medication to the experimental pharmacy for a pill count and subsequent destruction. They were also instructed to inform their GP and the researchers when they discontinued treatment earlier.

The participant’s own GP prescribed the study medication, guided treatment, monitored the patient’s safety, and provided usual care without any restrictions, both during and after treatment.

### Randomisation and blinding

Participants were randomly assigned and allocated to one of the three study arms: mirtazapine, amitriptyline, or placebo (ratio 1:1:1) using random sequence blocks (blocks of three). The blocks, generated by a computer algorithm, were stratified by the main type of sleep problem reported by the participants (that is, frequent waking versus waking up too early) under the responsibility of one of the authors.

During the treatment and follow-up period, randomisation was blinded for the research team, GPs, participants, and the participant’s community pharmacy. The research team remained blinded until the main effect analysis was performed. The author mentioned was not involved in data collection, and got involved after the completion of the blinded main effect analysis. GPs and participants were informed about the nature of the assigned treatment after the last participant had completed the final follow-up questionnaire.

### Outcomes

Patient-reported outcomes were assessed via online or paper-based questionnaires at baseline, during treatment at weeks 6 and 12, and during follow-up at weeks 20 and 52.^22^

The primary outcome was insomnia severity, as measured by the validated Insomnia Severity Index (ISI);^26^–^30^ this was measured at all predefined time points. The continuous ISI outcome at 6 weeks was the authors’ primary endpoint. The recall period was adapted from ‘the last month’ to ‘the past 2 weeks’, in alignment with other questionnaires and the option to increase the dosage up to week 4. In both the online and paper version only complete ISI questionnaires were included.

Secondary outcomes were:

- self-reported sleep quality (sleep indices): Pittsburgh Sleep Quality Index (PSQI) items 1–4,^31^ consensus sleep diary,^32^ and global rate of change (GRC);^33^
- daytime symptoms (fatigue [Multidimensional Fatigue Inventory {MFI}^34^,^35^ and Flinders Fatigue Scale {FFS} items 4 and 6^36^], anxiety and depression [Hospital Anxiety and Depression Scale {HADS}],^37^–^39^ and functioning [Work and Social Adjustment Scale {WSAS}^40^,^41^ and Glasgow Sleep Impact Index {GSII} parts 1, 2, and 3^42^]);
- treatment satisfaction;
- treatment sufficiency (additional sleep treatments during or after treatment);
- tolerability (side effects [Antidepressant Side-Effect Checklist {ASEC-21}^43^,^44^] and withdrawal symptoms [Discontinuation-Emergent Signs and Symptoms {DESS} checklist^45^,^46^]); and
- adherence.

Additional covariates included self-reported demographic and sleep characteristics at baseline, and information from the electronic medical record (EMR) held by the GP on the number of chronic diseases and sleep medication prescriptions in the year before baseline. Full wording of study specific questions are presented in the corresponding supplementary tables.

### Safety reporting

Serious adverse events and suspected unexpected serious adverse reactions were reported to the accredited medical ethics committee.^22^

### Sample size

A required sample size of 52 participants per study arm (156 in total) was calculated for the primary outcome (comparison of ISI score at 6 weeks between either amitriptyline or mirtazapine versus placebo).^22^

### Statistical analysis

The authors compared mirtazapine versus placebo and amitriptyline versus placebo. For the primary intention-to-treat (ITT) analyses, participants were analysed according to their randomisation group, regardless of whether they started and continued treatment. For the continuous outcomes, linear mixed-model analyses were performed. The standard model consisted of an analysis without the treatment variable, but with the interaction between the treatment variable and time.^47^ This model was used to adjust for baseline differences in the outcome between the groups. Time was treated as a categorical variable, represented by dummy variables, to estimate the differences between the groups at the different time points. Normality of the residuals was evaluated by visual inspection. For dichotomous outcomes, logistic generalised estimating equation (GEE) analyses^48^ were used and, for count data, Poisson mixed-model regression analyses were undertaken.

All linear mixed-model, logistic, and Poisson analyses were adjusted for the baseline value of the outcome, if available and applicable. As such, all models included all available values, took into account the dependency of repeated measurements within an individual, and were capable of handling missing data^49^ in a longitudinal dataset without the need to perform multiple imputations. In case an outcome was only measured on one time point, standard (linear, logistic, or Poisson) regression analyses were performed.

Descriptive statistics were used to describe treatment satisfaction, treatment sufficiency during treatment (additional sleep treatments), and adherence in the ITT group. For the primary outcome, the following were presented: the estimated mean difference, the proportion of participants that reported a clinically relevant improvement (ISI score of >7 points lower than baseline)^27^ or recovery (ISI score of ≤10),^27^ Cohen’s d,^50^ and the number needed to treat.^51^

Sleep diaries with at least 5 days completed were included in the analysis.^32^ Missing values on the MFI, HADS, and WSAS were replaced by the mean value of the participant’s completed items in the same scale when at least half of the items were completed. If fewer than half of the items were completed this questionnaire was not included in the analysis.^52^,^53^ Available minimal clinically important differences were accounted for while interpreting results.^33^,^34^,^37^,^41^

The presence of sleep medication prescriptions other than the study treatment were retrieved from the EMR for:

- the intended 16-week treatment period;
- the period participants reported to have actually been on treatment; and
- the 8 months following the intended treatment period.

For insomnia severity, secondary sleep and daytime outcomes, and body weight, the authors conducted similar secondary per protocol (PP) analyses including only those participants who:

- completed the full 16 weeks of treatment; and
- reported to have missed not more than 10 days of treatment.

Rebound insomnia, satisfaction with treatment duration, and additional sleep treatments after the study medication were described for the PP group only to evaluate the intended 16-week treatment.

A qualitative analysis of the GSII was performed across the entire sample.

Lastly, in *post-hoc* sensitivity analyses on the main outcome, the authors included those background variables in the model that turned out to be unequally distributed between the randomisation groups and that were associated with the main outcome variable — that is, sex and the presence of comorbidity.

For all statistical analyses, the two-sided significance level was set at *P*<0.05. SPSS Statistics for Windows (version 28.0) was used.

### Patient and public involvement

Independent (non-participating) patients with insomnia were consulted throughout the study: during the study set-up, this was done to assess the feasibility and completeness of relevant outcomes; after deblinding, it was done to explore their appraisal of the results.^22^

## Results

**Figure 1:**
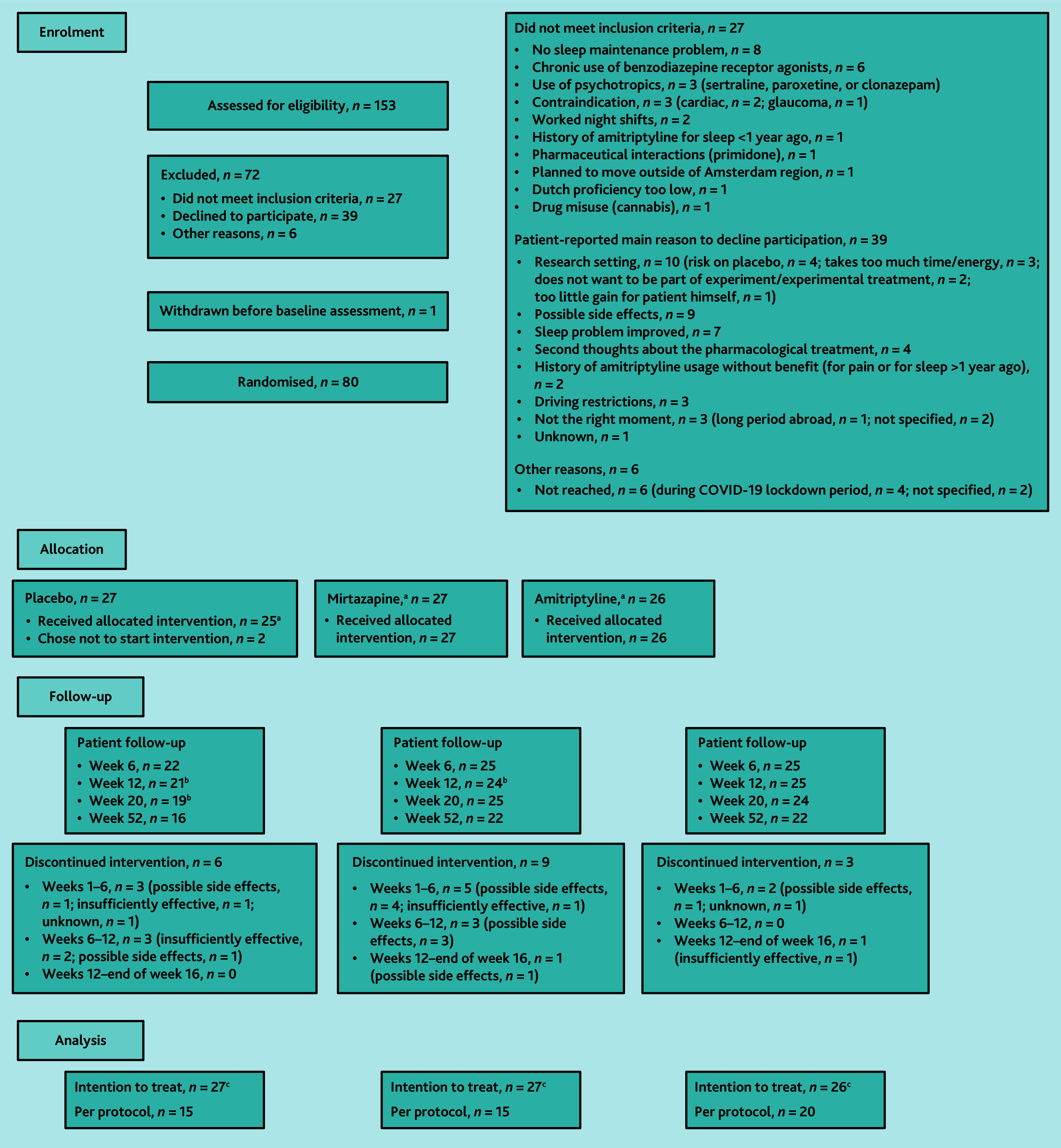
Flow of participants through the trial. ^a^16-week treatment with either mirtazapine (7.5–15 mg/day) or amitriptypine (10–20 mg/day), or placebo alongside usual care. Single dose at start, optional structural double-dose regime from week 2, 3, or 4 onwards and ending with single dose for last 2 weeks. ^b^Missed questionnaire. ^c^The primary outcome was complete in 90%, 88%, 85%, and 75% of the total group at 6, 12, 20, and 52 weeks’ follow-up.

Between January 2019 and June 2021, a total of 153 patients were assessed for eligibility, of which 80 participants were included and randomised (Figure 1).

The mean baseline ISI score was 19.0 (standard deviation [SD] = 3.9), indicating moderate insomnia. The randomisation groups were similar in terms of patient characteristics, except that the placebo group had a slightly higher percentage of females and subjects without comorbidity (see Supplementary Table S1).

### Treatment adherence

In total, 78 participants started treatment (Figure 1). On consultation with the GP at week 2, 3, or 4, 69 (88.5%) of the 78 participants received a follow-up prescription: single dose/double dose/no prescription was given to 17/6/4, 14/10/2, and 7/15/3 participants in the mirtazapine, amitriptyline, and placebo groups, respectively (see Supplementary Table S2). Three participants on double dose (one in each group) reported in the evaluation questionnaire that they had returned to structural single-dose regime. All in all, 60 (76.9%) patients completed the 16-week treatment (Figure 1). In 50 (64.1%) participants, treatment was considered as PP.

Based on pill count, >90% of the participants took pills on >80% of the treatment days. In total, 73.7% of patients in the placebo group reported that they thought they had received an active treatment or that they had no idea they were on a placebo, suggesting that blinding was successful.

### Effectiveness and tolerability of mirtazapine

**Figure 2:**
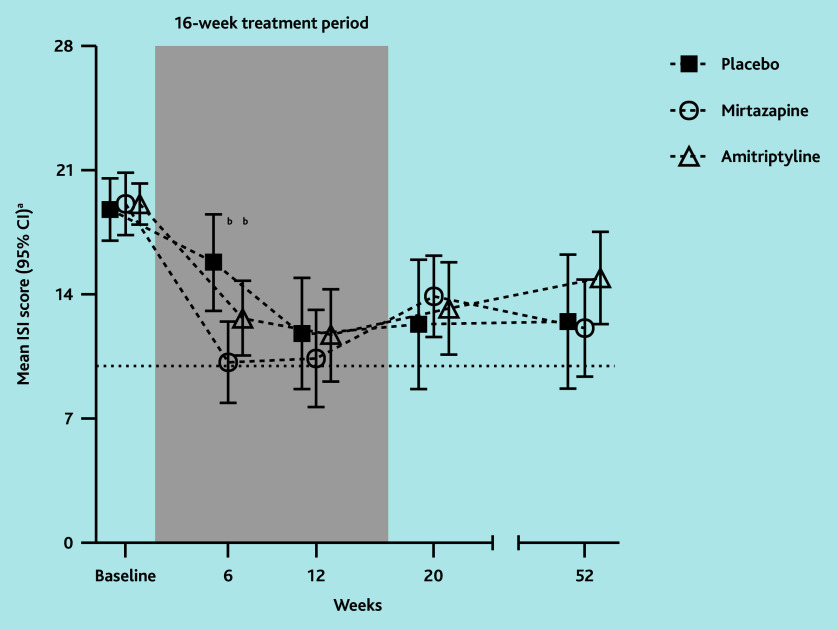
Mean ISI score based on ITT comparison between low-dose mirtazapine and amitriptyline, respectively, with placebo. ^a^ISI scores are interpreted as follows: absence of insomnia (0–7 points), sub-threshold insomnia (8–14 points), moderate insomnia (15– 21 points), and severe insomnia (22–28 points). Clinically relevant improvement = ISI score of >7 points lower than baseline.^27^ Recovery = ISI score of ≤10 points (horizontal dotted line and below) at follow-up. ^b^*P*<0.05; estimated mean difference significantly lower in mirtazapine compared with placebo and amitriptyline compared with placebo, respectively, based on ITT linear mixed-model analysis. ISI = Insomnia Severity Index. ITT = intention to treat.

In the ITT analyses, ISI scores in the mirtazapine group were statistically significantly lower than in the placebo group at week 6 (Figure 2). The Cohen’s *d* was 0.9 for ISI at 6 weeks (Table 1). At week 6, about half of the participants in the mirtazapine group reported clinically relevant improvement or recovery, which was statistically significantly more than in the placebo group. Although mean ISI scores and improvement and recovery rates in the mirtazapine group remained rather stable between week 6 and 12, from week 12–52 they were not statistically significantly different from those for placebo (in which 52.4% improved and 45.0% recovered at week 12).

**Table 1:** Primary outcome (ISI score) in the ITT group by placebo (*n* = 27), mirtazapine (*n* = 27), and amitriptyline (*n* = 26), and comparison between low-dose mirtazapine and amitriptyline, respectively, with placebo

Outcome measure^a^	Placebo	Mirtazapine	Amitriptyline	Mirtazapine versus placebo		Amitriptyline versus placebo	
**ISI score**	**Mean score (SD), ** ***n***	**Mean score (SD), ** ***n***	**Mean score (SD), ** ***n***	**Estimated mean difference (95% CI)**	**Cohen’s ** ***d*** b	**Estimated mean difference (95% CI)**	**Cohen’s ** ***d*** b
Baseline	18.8 (4.4), 27	19.1 (4.3), 27	19.1 (2.9), 26	—		—	—
Week 6	15.8 (6.2), 22	10.2 (5.6), 25	12.7 (5.1), 25	−6.0 (−9.0 to −3.0)^c^	0.9	−3.4 (−6.3 to −0.4)^c^	0.6
Week 12	11.8 (6.9), 21	10.4 (6.6), 24	11.7 (6.2), 25	−1.9 (−5.0 to 1.1)	—	−0.6 (−3.6 to 2.4)	—
Week 20	12.3 (7.6), 19	13.9 (5.6), 25	13.2 (5.9), 24	0.7 (−2.3 to 3.8)	—	0.2 (−2.9 to 3.2)	—
Week 52	12.5 (7.1), 16	12.1 (6.2), 22	14.9 (6.0), 22	−1.2 (−4.5 to 2.1)	—	1.8 (−1.5 to 5.0)	—

**Improved** d	***n*** **/** ***N*** ** (%)**	***n*** **/** ***N*** ** (%)**	***n*** **/** ***N*** ** (%)**	**OR (95% CI)**	**NNTB**	**OR (95% CI)**	**NNTB**
Week 6	3/22 (13.6)	13/25 (52.0)	10/25 (40.0)	6.9 (1.6 to 29.2)^c^	2.6 (1.6 to 7.1)	4.2 (1.0 to 18.1)	3.8 (2.0 to 41.7)
Week 12	11/21 (52.4)	13/24 (54.2)	11/25 (44.0)	1.1 (0.3 to 3.5)	—	0.7 (0.2 to 2.3)	—
Week 20	8/19 (42.1)	6/25 (24.0)	8/24 (33.3)	0.5 (0.1 to 1.8)	—	0.8 (0.2 to 2.7)	—
Week 52	6/16 (37.5)	11/22 (50.0)	6/22 (27.3)	1.8 (0.5 to 6.5)	—	0.6 (0.1 to 2.3)	—

**Recovery** e	***n*** **/** ***N*** ** (%)** f	***n*** **/** ***N*** ** (%)**	***n*** **/** ***N*** ** (%)**	**OR (95% CI)**	**NNTB**	**OR (95% CI)**	**NNTB**
Week 6	3/21 (14.3)	14/25 (56.0)	9/25 (36.0)	7.6 (1.8 to 32.7)^c^	2.4 (1.5 to 5.8)	3.4 (0.8 to 14.7)	4.6 (NNTH 43.0 to ∞ to NNTB 2.2)
Week 12	9/20 (45.0)	14/24 (58.3)	10/25 (40.0)	1.7 (0.5 to 5.5)	—	0.8 (0.3 to 2.8)	—
Week 20	7/18 (38.9)	7/25 (28.0)	7/24 (29.2)	0.7 (0.2 to 2.5)	—	0.7 (0.2 to 2.7)	—
Week 52	6/15 (40.0)	8/22 (36.4)	5/22 (22.7)	1.0 (0.3 to 4.0)	—	0.5 (0.1 to 2.2)	—

a For the continuous outcomes, linear mixed-model analyses were performed. For dichotomous outcomes, logistic generalised estimating equations analysis was used. Models included all measured values from all available time points.

b Effect expressed as SDs.^50^

c *P*<0.05.

d >7 points lower than baseline.

e Total score ≤10 points.

f One participant in the placebo group had an ISI score ≤10 at baseline and was therefore excluded from this analysis.

ISI = Insomnia Severity Index.^26^,^27^ ITT = intention to treat. NNTB = number needed to treat to benefit. NNTH = number needed to treat to harm.^51^ OR = odds ratio. SD = standard deviation.

Self-reported sleep duration was approximately 1 hour longer in the mirtazapine group than in the placebo group at weeks 6 and 12 (PSQI and sleep diary) (see Supplementary Table S3), and participants in the mirtazapine group were more likely to report sleep problems for <3 days per week at week 6. Smaller and less-consistent differences in favour of mirtazapine compared with placebo were seen:

- with sleep efficiency (on the sleep diary but not in the PSQI), and on the GRC, WSAS, and GSII at week 6;
- on the GRC and FFS at week 12; and
- with sleep efficiency at 52 weeks (see Supplementary Tables S3 and S4).

In the PP analysis, the differences compared with placebo found in the ITT analysis relating to ISI score (Table 2) and secondary outcomes (see Supplementary Tables S5 and S6) were more pronounced and often prolonged to week 12. In addition, a reduction in wake time after sleep onset (WASO) at week 6 and a clinically relevant reduction in anxiety, depression, and fatigue on all MFI subscales except motivation in week 12 was found. A *post-hoc* sensitivity analysis, adjusting for age and comorbidity, did not materially change the main results (see Supplementary Table S7).

**Table 2:** Primary outcome (ISI score) in the per protocol group^a^ by placebo (*n* = 15), mirtazapine (*n* = 15), and amitriptyline (*n* = 20), and comparison between low-dose mirtazapine and amitriptyline, respectively, with placebo

Outcome measure	Placebo	Mirtazapine	Amitriptyline	Mirtazapine versus placebo	Amitriptyline versus placebo
**ISI score**	**Mean score (SD), ** ***n***	**Mean score (SD), ** ***n***	**Mean score (SD), ** ***n***	**Estimated mean difference (95% CI)**	**Estimated mean difference (95% CI)**
Baseline	18.2 (4.8), 15	19.4 (3.9), 15	19.7 (2.6), 20	—	—
Week 6	15.7 (5.9), 15	8.1 (4.5), 15	13.2 (5.2), 20	−8.2 (−11.8 to −4.5)^b^	−3.2 (−6.6 to 0.2)
Week 12	12.7 (7.3), 15	7.2 (4.3), 15	11.6 (6.4), 20	−6.0 (−9.7 to −2.4)^b^	−1.8 (−5.1 to 1.6)
Week 20	14.3 (7.3), 15	12.7 (5.6), 15	13.2 (6.5), 20	−2.1 (−5.7 to 1.5)	−1.7 (−5.1 to 1.7)
Week 52	11.4 (7.3), 11	10.6 (6.9), 14	14.6 (6.4), 19	−1.5 (−5.5 to 2.4)	2.4 (−1.3 to 6.1)

**Improved** c	***n*** **/** ***N*** ** (%)**	***n*** **/** ***N*** ** (%)**	***n*** **/** ***N*** ** (%)**	**OR (95% CI)**	**OR (95% CI)**
Week 6	2/15 (13.3)	10/15 (66.7)	7/20 (35.0)	13.0 (2.1 to 81.5)^b^	3.5 (0.6 to 20.1)
Week 12	8/15 (53.3)	11/15 (73.3)	10/20 (50.0)	2.4 (0.5 to 11.1)	0.9 (0.2 to 3.3)
Week 20	4/15 (26.7)	5/15 (33.3)	7/20 (35.0)	1.4 (0.3 to 6.6)	1.5 (0.3 to 6.4)
Week 52	5/11 (45.5)	8/14 (57.1)	6/19 (31.6)	1.6 (0.3 to 7.2)	0.5 (0.1 to 2.2)

**Recovery** d	***n*** **/** ***N*** ** (%)** e	***n*** **/** ***N*** ** (%)**	***n*** **/** ***N*** ** (%)**	**OR (95% CI)**	**OR (95% CI)**
Week 6	2/14 (14.3)	10/15 (66.7)	6/20 (30.0)	12.0 (1.9 to 75.7)^b^	2.6 (0.4 to 15.2)
Week 12	5/14 (35.7)	11/15 (73.3)	8/20 (40.0)	5.0 (1.0 to 24.1)^b^	1.2 (0.3 to 4.9)
Week 20	3/14 (21.4)	5/15 (33.3)	7/20 (35.0)	1.8 (0.3 to 9.7)	2.0 (0.4 to 9.5)
Week 52	5/10 (50.0)	7/14 (50.0)	5/19 (26.3)	1.2 (0.3 to 5.8)	0.4 (0.1 to 1.9)

a On treatment for 16 weeks and reported no or <10 days of missed tablets. For the continuous outcomes, linear mixed-model analyses were performed. For dichotomous outcomes, logistic generalised estimating equations analysis was used. Models included all measured values from all available time points.

b *P*<0.05.

c >7 points lower than baseline.

d Total score = ≤10 points.

e One participant in the placebo group had an ISI score ≤10 at baseline and therefore was excluded from this analysis.

ISI = Insomnia Severity Index.^26^,^27^ OR = odds ratio. SD = standard deviation.

Mirtazapine was generally tolerated and evaluated well (see Supplementary Tables S8–S11); however, compared with the placebo group, side effects and treatment discontinuation because of side effects were more common in the mirtazapine group, although this last difference was not statistically significant. No statistically significant differences in self-reported body weight during or after treatment were found (see Supplementary Table S8).

### Effectiveness and tolerability of amitriptyline

In the ITT analyses, ISI scores in the amitriptyline group were statistically significantly lower than in the placebo group at week 6, but this difference was not clinically relevant. The Cohen’s *d* was 0.6 for ISI at 6 weeks. No statistically significant differences compared with placebo were seen for improvement or recovery rates or at later time points (Table 1 and Figure 2). In addition, no statistically significant and clinically relevant improvements were found in relation to sleep and daytime outcomes during and after treatment when compared with placebo (see Supplementary Tables S3 and S4). Differences in favour of amitriptyline on GRC at week 6 and 12 and WSAS at week 6 were smaller than clinically relevant. In contrast, the amitriptyline group had less-favourable scores on overall fatigue and physical fatigue (MFI) at 20 weeks compared with the placebo group, which exceeded the threshold for clinical relevance.

In the PP analysis, ISI scores at 6 weeks were no longer statistically significantly different from placebo (Table 2). However, the amitriptyline group had clinically relevant more-favourable GRC scores at week 6 and 12, and a HADS depression score at week 12 compared with placebo (see Supplementary Tables S5 and S6). The *post-hoc* sensitivity analyses did not materially change the main results (see Supplementary Table S7). Amitriptyline was generally tolerated and evaluated well (see Supplementary Tables S8–S11).

### Treatment sufficiency

Three participants in each group received ≥1 prescription for sleep medication other than the study medication in the weeks these participants reported to have actually been on treatment (see Supplementary Table S12). In the double-blind 8 months after treatment, half (*n* = 25) of the participants in the PP group received a subsequent sleep medication prescription, most often either low-dose amitriptyline or mirtazapine.

### Other outcomes

Qualitative analysis of the GSII across the entire sample showed that, for participants at baseline, the main impairments due to their sleep problem were in the domains of emotional regulation, energy/motivation, and performance at daily activities (see Supplementary Table S13).

### Safety reporting

In total, two serious adverse events concerning one participant were reported; these were admissions to hospital that were judged not to be related to the intervention.

## Discussion

### Summary

This placebo-controlled study in 80 patients with insomnia disorder in general practice demonstrated a statistically significant and clinically relevant effect of low-dose mirtazapine (7.5–15 mg/day) on insomnia severity at 6 weeks, but not at 12 weeks during treatment or at follow-up to 12 months. Secondary sleep outcomes and the PP analysis supported effectiveness of low-dose mirtazapine up to at least 6 weeks. Side effects and discontinuation due to side effects were more common in the mirtazapine group compared with placebo, suggesting lower tolerability. However, this last difference was not statistically significant.

Low-dose amitriptyline (10–20 mg/day) resulted in a statistically significant reduction in insomnia severity at week 6 only, which was smaller than clinically relevant. There were no statistically significant and clinically relevant improvements on secondary outcomes for amitriptyline during treatment or follow-up to 12 months. Amitriptyline was tolerated well.

### Strengths and limitations

Strengths of the study are the fact that, to the authors’ knowledge, it is the first placebo-controlled study on this subject, along with its long follow-up, the comprehensive assessment of both insomnia severity and daytime consequences, and its placebo-controlled and pragmatic design, which reflected common off-label treatment and contributes to the generalisability of the results. Furthermore, in this ambulant study >90% of the participants who started treatment took their study medication on >80% of the treatment days.

The main limitation concerns the low study sample size, followed by the authors only including 51.0% of the targeted number (*n* = 156) of patients in the study’s available time and resources. This was mainly due to the COVID-19 pandemic restrictions and their impact on primary care.^54^ Despite the lower power, statistically significant differences compared with placebo were found relating to insomnia severity for both amitriptyline and mirtazapine at 6 weeks. Given the marginal effect for amitriptyline, it is likely that a larger number of participants would not have led to a clinically relevant effect. As mirtazapine demonstrated larger and clinically relevant effects on both insomnia severity and secondary outcomes, the authors expect their conclusions regarding short-term effectiveness to hold with a larger sample size. However, the lower number might have affected the results at later time points and the PP analysis, even though the loss to follow-up rate was lower than accounted for.^22^ The planned explorative subgroup analyses (for example, dosage, sex, and other background characteristics)^22^ were not conducted because of the limited sample size.

### Comparison with existing literature

The findings suggest that the effect of mirtazapine on insomnia severity in the short term is large. For comparison, previously standardised mean differences on subjective sleep quality compared with placebo up to 0.83 have been reported for short-acting benzodiazepines at 4 weeks,^21^ and up to 0.4 for low-dose doxepin, trimipramine, trazodone, mianserin, or combinations of them.^14^,^21^ In a network meta-analysis of pharmacological interventions for insomnia, improvement rates on subjective sleep quality measured by a sleep index were up to 30% for short-acting benzodiazepine receptor agonists, 20% for doxepin, and 24% for trazodone at ~4 weeks, compared with 16% in the placebo group.^21^

The data presented here did not support the long-term effectiveness of mirtazapine that had previously been reported in a retrospective, non-controlled, cross-sectional, chart study^18^ among patients with chronic insomnia treated with low-dose mirtazapine in a sleep disorder centre. In that study, the majority of participants no longer met the criteria for insomnia at the end of the treatment period of 3–6 months; this comprised 87% of the 46 patients who were on treatment for at least 3 months, a figure that fell to 66% when 21 additional patients who were lost to follow-up before 3 months were included.^18^ In the trial presented here, with a randomised placebo-controlled design and a primary care population, insomnia severity did not significantly differ from placebo at 12 weeks. It should be noted that the increase of sleep duration compared with placebo was large and still statistically significant at 12 weeks; similar increases in total sleep time were reported for low-dose esmirtazapine in industry-funded trials.^55^–^58^

Contrary to the authors’ expectations,^20^,^22^ improvements in sleep maintenance parameters, such as WASO or a consistent increase in sleep efficiency, were not found.

All-cause discontinuation for mirtazapine in the trial (9 out of 27 participants before 16 weeks) was in line with real-world data, such as the above-mentioned chart study^18^ and an earlier usual care study^13^ in which a third of participants did not receive a repeat prescription for mirtazapine.

In an open-label amitriptyline study^19^ by the authors of the present study, a larger proportion of participants reported improvement (sleep maintenance improved in 74%) around week 6, while in the trial 40% had clinically relevant improvement on the ISI. The difference between the two studies could be due to the difference between primary care and secondary care populations. However, it could also be due to the difference in how ‘improvement’ was defined. A *post-hoc* examination of the trials’ GRC scores also revealed that, at 6 weeks in the trial population, 72% of the participants on amitriptyline experienced ‘some improvement’ in their sleep problem compared with baseline and 28% no change (versus 52% improvement, 19% no change, and 29% worsening in the placebo group). In both the open-label study and the trial, 54% of the participants remained on a single dose; 46% reported being (very) satisfied with the treatment results (assessed at week 6 and week 20, respectively), and <5% discontinued treatment early.^19^

The present study used a placebo group as reference and was not designed to detect differences between amitriptyline and mirtazapine; nevertheless, the statistical analysis applied enabled a *post-hoc* comparison between the results of the groups randomised to these two drugs. No statistically significant difference on the primary outcome was observed (continuous ISI score at 6 weeks) (*P* = 0.07). The slightly smaller effect of amitriptyline combined with its larger tolerability raises the question whether this could be a consequence of relatively lower investigated dosages of amitriptyline, rather than of intrinsic differences between the drugs. Dosage was, indeed, increased a little more often for amitriptyline than for mirtazapine. On the other hand, in both groups the majority of participants remained on a single dosage, and in the amitriptyline group not more often extra tablets than agreed on with the GP were taken in comparison with the other groups.

Improvements in the placebo group were observed over time. It has been reported that a placebo response on subjective insomnia outcomes is common; this consists of physiological changes produced by using a placebo, the non-specific effects of participating in a trial, natural disease course, and regression to the mean.^59^ The placebo response in the study presented here, with 52% of participants reporting improvement on ISI at week 12, is larger than in the combined data from >12 000 patients randomly assigned to placebo in sleep medication trials, in which only 16% improved in the short term (4 weeks) and 25% in the long term (>12 weeks).^21^ The placebo response is also large compared with the ISI improvement rates of a care-as-usual/waiting list control group in a randomised controlled trial on CBT-I in a similar Dutch general practice population in which 12% improved at week 8 and 11% at week 26 follow-up.^60^ Possible explanations for a larger placebo response are the pragmatic design of the study presented here without a placebo run-in and because the participants may well have visited their GP when they were experiencing severe complaints. The data presented here did not show a larger use of additional sleep treatments in the placebo group or a large loss to follow-up between weeks 6 and 12 compared with the other groups.

### Implications for research and practice

Based on the present study, the prescription of off-label low-dose mirtazapine or amitriptyline to patients with insomnia disorder and sleep maintenance problems, who request daily sleep medication in general practice, is not recommended if the aim is to achieve sustained sleep improvement. When quick improvement is desired, off-label low-dose mirtazapine might be considered for a period up to 6–12 weeks. Monitoring through follow-up consultations is important for evaluating side effects versus intended effect and timely discontinuation of treatment. Future research in a larger sample is warranted to ascertain whether the results presented here would be replicated, study the optimal treatment duration, and assess the risk:benefit ratio in relation to other treatments.

## Supplementary Information



## Data Availability

Study protocol and statistical analysis plans will be available. Data sharing of aggregated or anonymised participant data for scientific purposes within the informed consent of the study participants is part of the obligatory data management protocol of the funding agency. Any requests can be sent to the corresponding author.
