# Supplementary material for: Effectiveness of low-dose amitriptyline and mirtazapine in patients with insomnia disorder and sleep maintenance problems: a randomised, double-blind, placebo-controlled trial in general practice (DREAMING)
Source: Br J Gen Pract. 2025 Jun 17;75(756):e474–83. doi: 10.3399/BJGP.2024.0173 (PMC12199994; doi:10.3399/BJGP.2024.0173)
Supplement: Supplementary file 1 [file BJGP.2024.0173_suppl.pdf]

Supplementary files

Box S1. Eligibility and consent procedure, selection criteria DREAMING study

Eligibility was assessed in three subsequent checks. Firstly, when a patient requested sleep medication, the patient's own GP checked upon consultation whether medication was indicated and whether amitriptyline or mirtazapine was not contraindicated or would pose additional risks (criteria 1–15) based on the information known to the GP. If applicable, they obtained written permission from the patient to be contacted by the researchers. Secondly, during a phone call the researcher verified (scientific) eligibility (criteria 1, 8 and 13–16), assessed the patients main type of sleep problem for stratification purposes (i.e. frequent wakening vs waking up too early), and scheduled a follow up meeting to obtain informed consent. Thirdly, the pharmacist of the experimental pharmacy checked medication safety (criterion 10) based on the self-reported use of over-the-counter remedies and a list of currently used prescription medication from the participants' community pharmacy. Upon written consent, the researchers provided the baseline questionnaire and agreed on the study medication start date (t= week 1).

Inclusion criteria were (1) insomnia disorder, clinically assessed in general practice based on the Diagnostic and Statistical Manual of Mental Disorders-5 (DSM-5) criteria (1); (2) Non-pharmacological treatment according to the Dutch general practice guideline (including sleep hygiene advice and cognitive behavioural approaches) is deemed insufficient by patient and general practitioner (GP); (3) Consultation of the GP for a sleep medication request, other than for occasional incidental nights or specific period (e.g., travelling); (4) Aged between 18 and 85 years; (5) Enlisted as patient in one of the participating general practices during the treatment and safety monitoring period.
Exclusion criteria were (6) Insomnia secondary to another medical condition, for example, obstructive sleep apnea syndrome (OSAS), comorbid major depression, chronic pain; (7) Amitriptyline or mirtazapine is contraindicated or would pose additional risks, that is, known allergy for amitriptyline or mirtazapine, cardiac arrhythmia/ cardiac blockade/long QT syndrome/Brugada syndrome/family history of acute cardiac death/recent myocardial infarction (within the past 90 days)/angina pectoris/coronary insufficiency, severe renal insufficiency (Glomerular Filtration Rate, GFR <10), severe liver dysfunction, epilepsy, ocular hypertension/glaucoma, bipolar affective disorder, concurrent alcohol or drug abuse/addiction, suicide risk, vulnerability due to known unstable health situation, according to GP; (8) Pregnancy, lactation or wish to become pregnant in the next 6 months; (9) Terminal illness; (10) Potential drug–drug interactions: chronic use of psychotropic drugs (including anxiolytics, antidepressants, antipsychotics and anticonvulsants and stimulants, note: Incidental use of BZRAs for sleep in the preceding months is allowed), concurrent use of oral antimycotics, enzyme inductors, antiretroviral drugs, cimetidine and clonidine; (11) prescription of amitriptyline or mirtazapine for insomnia in the past year; (12) Being unable to follow study instructions and fill out the study questionnaires (in Dutch); (13) Isolated sleep initiation problem (i.e., without problems maintaining sleep or early-morning awakening problems); (14) Doing night shifts on a regular basis; (15) Wish to continue (over-the-counter) sleep aids containing melatonin, St John's worth, cannabis or antihistamines; (16) Concurrent participation in clinical intervention study interfering with the DREAMING intervention and study procedures.

Table S1. Baseline background and clinical characteristics of participants of the DREAMING study, total intention-to-treat sample, randomly assigned to placebo, low-dose mirtazapine or low-dose amitriptyline. Values are numbers (column percentages) unless stated otherwise.

	Total randomised (n =80)	Placebo (n=27)	Mirtazapine (n=27)	Amitriptyline (n=26)
Patient characteristics				
Mean (SD) age (years), number	50.1 (16.2), 80<br>(min-max 20-83)	48.1 (16.5), 27	51.7 (15.8), 27	50.5 (16.8), 26
18-25<br>26-40<br>41-65<br>66-85	3 (3.8)<br>23 (28.7)<br>39 (48.8)<br>15 (18.8)	0 (0.0)<br>10 (37.0)<br>14 (51.9)<br>3 (11.1)	2 (7.4)<br>5 (18.5)<br>14 (51.9)<br>6 (22.2)	1 (3.8)<br>8 (30.8)<br>11 (42.3)<br>6 (23.1)
Female sex<br>Male sex	58 (72.5)<br>22 (27.5)	22 (81.5)<br>5 (18.5)	19 (70.4)<br>8 (29.6)	17 (65.4)<br>9 (34.6)
Mean (SD) BMI, number	23.4 (4.7), 78	25.6 (6.4), 27	23.7 (2.9), 27	23.7 (4.0), 26
Level of education<br>Higher (vocational or university)<br>Lower, middle and other<br>Missing	 49 (61.3)<br>29 (36.3)<br>2 (2.5)	 16 (59.3)<br>11 (40.7)<br>0 (0.0)	 16 (59.3)<br>10 (37.0)<br>1 (3.7)	 17 (65.4)<br>8 (30.8)<br>1 (3.8)
Working status<br>Paid job or own company<br>Other*<br>Missing	 47 (58.8)<br>27 (33.8)<br>6 (7.5)	 16 (59.3)<br>9 (33.3)<br>2 (7.4)	 14 (51.9)<br>10 (37.0)<br>3 (11.1)	 17 (65.4)<br>8 (30.8)<br>1 (3.8)
Number of chronic diseases† registered at baseline (EMR data)<br>0<br>1<br>2<br>3 or more	 33 (41.3)<br>15 (18.8)<br>13 (16.3)<br>19 (23.8)	 13 (48.1)<br>5 (18.5)<br>3 (11.1)<br>6 (22.2)	 10 (37.0)<br>5 (18.5)<br>6 (22.2)<br>6 (22.2)	 10 (38.5)<br>5 (19.2)<br>4 (15.4)<br>7 (26.9)
Current smoking status<br>No<br>Yes (daily or on occasions)<br>Missing	 60 (75.0)<br>14 (17.5)<br>6 (7.5)	 19 (70.4)<br>6 (22.2)<br>2 (7.4)	 20 (74.0)<br>4 (14.8)<br>3 (11.1)	 21 (80.8)<br>4 (15.4)<br>1 (3.8)
Alcohol consumption in the past 6 month<br>Rarely or none; less than 1 day per week<br>1-7 days a week<br>Missing	 36 (45.0)<br>37 (46.3)<br>7 (8.8)	 11 (40.7)<br>13 (48.1)<br>3 (11.1)	 12 (44.4)<br>12 (44.4)<br>3 (11.1)	 13 (50.0)<br>12 (46.2)<br>1 (3.8)

**Table S1.** Continued				
Sleep related characteristics				
Mean (SD) duration of insomnia (years), number	13.0 (14.4), 80	14.3 (15.2), 27	11.2 (15.1), 27	13.5 (13.1), 26
Main type of sleep problem as assessed by the researchers during phone call§				
Waking up too early in the morning or at night and trouble falling asleep again(EMA)	36 (45.0)	11 (40.7)	13 (48.1)	12 (46.2)
Frequent wakening during the night (Sleep maintenance insomnia)	44 (55.0)	16 (59.3)	14 (51.9)	14 (53.8)
Self-reported chronotype				
Morningness	23 (28.7)	7 (25.9)	8 (29.6)	8 (30.8)
Eveningness	18 (22.5)	4 (14.8)	7 (25.9)	7 (26.9)
None of both	31 (38.8)	12 (44.4)	9 (33.3)	10 (38.5)
Missing	8 (10.0)	4 (14.8)	3 (11.1)	1 (3.8)
Mean (SD) self-estimated sleep need (in hours per night), number	7.1 (1.1), 72	7.1 (1.0), 23	7.3 (0.9), 24	6.8 (1.3), 25
Sleep medication prescription in the year before baseline (EMR data)				
No	40 (50.0)	16 (59.3)	14 (51.9)	10 (38.5)
Yes	40 (50.0)	11 (40.7)	13 (48.1)	16 (61.5)
Type of sleep medication (multiple answers possible):				
BZRA	35 (43.8)	10 (37.0)	10 (37.0)	15 (57.7)
Melatonin	4 (5.0)	1 (3.7)	3 (11.1)	0 (0.0)
Other	1 (1.3)	0 (0.0)	0 (0.0)	1 (3.8)
Ever used sleep aids (prescribed or over the counter (OTC) self-reported				
Yes	67 (82.7)	24 (88.9)	21 (77.8)	22 (84.6)
No	13 (16.3)	3 (11.1)	6 (22.2)	4 (15.4)
Type of sleep aid:				
BZRA, (almost) daily	19 (23.8)	6 (22.2)	6 (22.2)	7 (26.9)
BZRA, if needed	43 (53.8)	14 (51.9)	11 (40.7)	18 (69.2)
Amitriptyline/Mirtazapine	2 (2.5)	0 (0.0)	1 (3.7)	1 (3.8)
Melatonin (prescribed or OTC)	35 (43.8)	13 (48.1)	13 (48.1)	9 (34.6)
Other over the counter treatment	35 (43.8)	10 (37.0)	13 (48.1)	12 (46.2)
Specification missing	2 (2.5)	0 (0.0)	1 (3.7)	1 (3.8)
Ever had course or non-pharmacological therapy for sleep‡				
No	58 (72.5)	21 (77.8)	20 (74.1)	17 (65.4)
Yes	22 (27.5)	6 (22.2)	7 (25.9)	9 (34.6)

*SD: standard deviation; BMI: body mass index; EMR: electronic medical records in general practice; BZRA: benzodiazepine receptor agonist; OTC: over the counter remedy. *Other employment status included retired, unemployed, incapacitated, on social security, volunteer work/family caregiver, housewife/husband, in education/studying. † History of cardiovascular disease, hypertension, diabetes, kidney damage, asthma/pulmonary emphysema, chronic diseases of the locomotor apparatus associated with pain, chronic neurological diseases including headache syndromes, inflammatory bowel disease, thyroid problem, history of any malignancy. malignancy, based on data from the electronic medical record in general practice. ‡By a neurologist/somnologist, mental health nurse practitioner, psychologist, sleep therapist, (online) self-help course, or other type of course or therapy. § I would now like to ask you what kind of sleeping problems you have and also what bothers you the most. Do you suffer from (i) waking up frequently during the night, after which you fall asleep again after some time? (ii) waking up and not falling back asleep during the night? (iii) waking up too early in the morning? If multiple, what bothers you the most?*

Table S2. Secondary sleep outcomes in the intention-to-treat group per treatment group, comparison between low dose mirtazapine and amitriptyline respectively with placebo. Values are mean score (SD), number, unless stated otherwise

Outcome measure at each measurement time point	Placebo	Mirtazapine	Amitriptyline	Mirtazapine vs Placebo	Amitriptyline vs placebo
Intention to treat analysis	N=27	N=27	N=26		
PSQI					
TST (hours)				Estimated mean difference (95% CI)	
Baseline	4.7 (1.1), 26	4.8 (1.1), 26	5.0 (1.2), 26	-	-
Week 6	5.5 (1.1), 19	6.4 (1.0), 25	6.0 (1.6), 25	0.9 (0.2 to 1.6)*	0.4 (-0.3 to 1.0)
Week 12	5.7 (1.9), 21	6.6 (1.3), 23	6.3 (1.3), 25	1.0 (0.3 to 1.6)*	0.5 (-0.1 to 1.2)
Week 20	5.7 (1.9), 19	5.9 (1.2), 25	5.9 (1.4), 24	0.2 (-0.4 to 0.9)	0.1 (-0.5 to 0.8)
Week 52	5.4 (1.9), 15	6.2 (1.5), 22	6.0 (1.4), 22	0.9 (0.2 to 1.6)	0.5 (-0.2 to 1.3)
SOL (minutes)				Estimated mean difference (95% CI)	
Baseline	69.7 (72.3), 27	34.9 (33.7), 26	53.4 (37.7), 26	-	-
Week 6	57.4 (62.9), 22	24.3 (15.7), 25	68.0 (95.3), 25	-18.1 (-46.3 to 10)	18.2 (-9.9 to 46.3)
Week 12	48.0 (46.1), 20	35.2 (34.1), 23	52.5 (56.2), 25	-4.9 (-34.1 to 24.4)	5.6 (-23.2 to 34.3)
Week 20	34.8 (26.3), 19	44.8 (49.9), 24	43.6 (39.3), 24	15.7 (-13.7 to 45.0)	4.4 (-24.9 to 33.7)
Week 52	72.1 (91.1), 14	32.7 (32.0), 22	43.8 (53.8), 22	-26.3 (-58.5 to 5.9)	-22.1 (-54.3 to 10)
SE (%)				Estimated mean difference (95% CI)	
Baseline	55.4 (16.9), 25	61.0 (24.8), 26	60.0 (14.1), 26	-	-
Week 6	65.2 (18.9), 19	74.0 (14.4), 25	66.1 (17.9), 25	6.6 (-2.0 to 15.2)	-2.1 (-10.7 to 6.4)
Week 12	74.0 (27.5), 20	74.7 (17.6), 23	69.4 (18.0), 25	4.2 (-4.4 to 12.7)	-3.1 (-11.5 to 5.4)
Week 20	74.0 (19.6), 18	66.8 (15.5), 24	68.0 (15.8), 24	-3.7 (-12.5 to 5.0)	-2.9 (-11.6 to 5.8)
Week 52	61.2 (25.1), 15	71.9 (17.2), 22	68.7 (14.9), 22	9.9 (0.7 to 19.1)*	4.4 (-4.8 to 13.6)
Frequency of sleep problem; 3 or more days per week (No(%), n)				Odds ratio (95% CI)	
Baseline	23 (92.0), 25	25 (96.2), 26	25 (96.2), 26	-	-
Week 6	15 (75.0), 20	10 (40.0), 25	15 (60.0), 25	0.2 (0.1 to 0.8)*	0.5 (0.1 to 1.8)
Week 12	11 (52.4), 21	7 (30.4), 23	15 (62.5), 24	0.3 (0.1 to 1.2)	1.3 (0.4 to 4.2)
Week 20	9 (50.0), 18	16 (64.0), 25	14 (58.3), 24	1.4 (0.4 to 4.8)	1.1 (0.3 to 3.7)
Week 52	12 (75.0), 16	13 (59.1), 22	15 (68.2), 22	0.4 (0.1 to 1.8)	0.7 (0.1 to 3.1)

**Table S2.** Continued					
Consensus Sleep Diary					
TST (hours)				Estimated mean difference (95% CI)	
Baseline<br>Week 6<br>Week 20	5.4 (1.2), 23<br>5.9 (1.3), 21<br>5.8 (1.6), 16	5.2 (1.2), 23<br>6.9 (1.1), 22<br>6.0 (1.3), 20	5.7 (1.0), 21<br>6.6 (1.4), 19<br>6.5 (1.4), 18	-<br>1.2 (0.6 to 1.8)*<br>0.5 (-0.1 to 1.1)	-<br>0.5 (-0.1 to 1.0)<br>0.4 (-0.2 to 1.1)
SOL (minutes)				Estimated mean difference (95% CI)	
Baseline<br>Week 6<br>Week 20	88.0 (74.7), 23<br>67.2 (61.0), 21<br>77.1 (109.7), 16	42.3 (43.8), 23<br>32.6 (28.9), 22<br>51.4 (39.8), 20	50.0 (29.9), 21<br>61.0 (61.7), 19<br>43.4 (43.8), 18	-<br>-16.4 (-48.2 to 15.3)<br>-12.8 (-47.3 to 21.8)	-<br>13.8 (-19.1 to 46.7)<br>-19.2 (-54.6 to 16.1)
SE (TST/TIB*100%)				Estimated mean difference (95% CI)	
Baseline<br>Week 6<br>Week 20	61.8 (15.8), 23<br>69.7 (17.2), 21<br>66.3 (14.5), 16	62.0 (13.8), 23<br>76.1 (12.1), 22<br>66.1 (11.8), 20	67.4 (11.6), 21<br>73.4 (13.8), 19<br>74.0 (15.1), 18	-<br>8.3 (1.6 to 14.9)*<br>1.5 (-5.7 to 8.7)	-<br>0.2 (-6.7 to 7.1)<br>3.2 (-4.2 to 10.6)
WASO (minutes)				Estimated mean difference (95% CI)	
Baseline<br>Week 6<br>Week 20	121.3 (72.2), 23<br>96.5 (57.2), 21<br>100.3 (52.6), 16	151.9 (58.8), 23<br>98.1 (59.0), 22<br>133.6 (74.8), 20	118.0 (63.1), 21<br>85.5 (51.1), 19<br>96.4 (57.6), 18	-<br>-25.0 (-54.4 to 4.4)<br>11.9 (-19.9 to 43.6)	-<br>-14.4 (-44.9 to 16.1)<br>4.2 (-28.3 to 36.8)
NWAK				Estimated mean difference (95% CI)	
Baseline<br>Week 6<br>Week 20	1.2 (0.9), 23<br>1.2 (0.9), 21<br>1.4 (0.8), 16	1.3 (0.7), 23<br>1.3 (0.7), 22<br>1.4 (0.7), 20	1.2 (0.7), 21<br>1.1 (0.8), 19<br>1.0 (0.8), 18	-<br>0.0 (-0.4 to 0.4)<br>0.0 (-0.4 to 0.4)	-<br>-0.1 (-0.5 to 0.3)<br>-0.2 (-0.6 to 0.3)
TWAK (minutes)				Estimated mean difference (95% CI)	
Baseline<br>Week 6<br>Week 20	48.8 (50.3), 23<br>34.2 (32.7), 21<br>35.5 (34.8), 16	62.1 (47.6), 23<br>37.2 (29.5), 22<br>41.5 (33.0), 20	47.5 (35.4), 21<br>39.7 (27.4), 19<br>46.9 (41.8), 19	-<br>-5.5 (-25.7 to 14.8)<br>1.0 (-21.0 to 23.1)	-<br>5.8 (-15.2 to 26.8)<br>15.9 (-6.7 to 38.5)
Sleep Quality (1-10)				Estimated mean difference (95% CI)	
Baseline<br>Week 6<br>Week 20	5.0 (1.7), 18<br>6.3 (1.5), 18<br>5.6 (1.9), 13	4.7 (1.2), 22<br>6.5 (1.3), 20<br>5.2 (1.0), 18	5.2 (0.8), 20<br>6.3 (1.2), 16<br>6.0 (1.7), 15	-<br>0.5 (-0.3 to 1.3)<br>0.0 (-0.8 to 0.9)	-<br>0.1 (-0.7 to 0.9)<br>0.5 (-0.4 to 1.4)
Refreshing Quality of Sleep (1-10)				Estimated mean difference (95% CI)	
Baseline<br>Week 6<br>Week 20	5.0 (1.8), 17<br>6.0 (1.4), 21<br>6.0 (1.9), 13	4.9 (1.2), 21<br>6.3 (1.0), 20<br>5.5 (1.0), 18	5.3 (1.0), 20<br>6.2 (1.1), 17<br>6.0 (1.5), 15	-<br>0.4 (-0.3 to 1.2)<br>-0.3 (-1.2 to 0.6)	-<br>0.1 (-0.7 to 0.9)<br>0.1 (-0.8 to 1.0)

**Table S2.** Continued					
Global rate of change (-5 to 5)				Estimated mean difference (95% CI)	
Week 6	0.6 (2.5), 21	2.4 (1.6), 25	1.8 (1.4), 25	1.8 (0.6 to 3.0)*	1.2 (0.0 to 2.4)*
Week 12	0.8 (2.8), 20	2.4 (2.0), 23	2.0 (1.9), 24	1.6 (0.4 to 2.8)*	1.3 (0.1 to 2.5)*
Week 20	1.2 (2.5), 18	1.4 (1.7), 25	1.0 (2.2), 23	0.5 (-0.7 to 1.7)	0.0 (-1.3 to 1.2)

*SD: standard deviation. Vs: versus. PSQI: Pittsburgh Sleep Quality Index (31), item 1-4, recall period adapted to the past 2 weeks, assessed at all time points. TST: Total Sleep Time, hours. CI: confidence interval. SOL: Sleep Onset Latency, minutes, number of minutes it takes to fall asleep. SE: sleep efficiency, 0-100%, total sleep time divided by time between lights out time and final rise time *100%. Frequency of sleep problem: reported number of nights per week experiencing sleep problem, % reporting on 3 or more nights per week, assessed at all time points. Consensus sleep diary(32): prospectively filled out over one week at baseline, week6 and 20. TIB: time in and out bed, hours, time between lights out time and final rise time. WASO: Wake time After Sleep Onset, minutes, total minutes awake after sleep onset and before final rise time regardless whether it was spend in or out bed. NWAK: Number of awakenings, number of night-time awakenings per night. TWAK: terminal wakefulness, minutes, time between final awakening and final rise time. Sleep Quality: sleep quality score, scale 1-10, higher score favourable. Refreshing Quality of Sleep: refreshing Quality of Sleep score, scale 1-10, higher score favourable. Global rate of change (GRC) (33): scale -5 to 5, assessing to what extent the sleep problem was experienced as being improved, worsened or unchanged compared to baseline; higher score favourable, MCID 2 points, assessed at week 6, 12 and 20.*

** p <0.05. For the continuous outcomes linear mixed model analyses were performed. For dichotomous outcomes, logistic generalized estimating equations (GEE) analysis was used. Models included all measured values from all available time points.*

Table S3. Secondary daytime outcomes in the intention-to-treat group per treatment group, comparison between low dose mirtazapine and amitriptyline respectively with placebo. Values are mean score (SD), number, unless stated otherwise

Outcome measure at each measurement time point	Placebo	Mirtazapine	Amitriptyline	Mirtazapine vs Placebo	Amitriptyline vs placebo
Intention to treat analysis	N=27	N=27	N=26		
MFI					
general fatigue (4-20)				Estimated mean difference (95% CI)	
Baseline	16.9 (3.4), 27	17.3 (3.1), 27	16.0 (2.4), 26	-	-
Week 12	12.7 (4.0), 20	12.6 (4.7), 22	13.6 (4.4), 24	-0.4 (-2.5 to 1.8)	1.0 (-1.1 to 3.2)
Week 20	11.4 (4.0), 18	13.6 (4.1), 24	14.1 (4.5), 21	1.8 (-0.4 to 4.0)	2.6 (0.4 to 4.9)*
physical fatigue (4-20)				Estimated mean difference (95% CI)	
Baseline	14.8 (3.6), 27	14.5 (4.6), 27	13.9 (2.9), 26	-	-
Week 12	11.8 (4.5), 20	10.9 (4.3), 22	11.9 (4.5), 23	-0.6 (-2.7 to 1.6)	0.5 (-1.6 to 2.6)
Week 20	10.6 (3.4), 18	11.5 (4.2), 24	13.2 (4.5), 19	0.7 (-1.5 to 2.9)	2.6 (0.3 to 4.8)*
reduced activity (4-20)				Estimated mean difference (95% CI)	
Baseline	14.4 (3.6), 26	14.4 (4.1), 24	13.0 (3.5), 25	-	-
Week 12	11.8 (4.2), 20	10.5 (4.6), 20	12.3 (4.1), 23	-1.4 (-3.6 to 0.8)	1.0 (-1.1 to 3.1)
Week 20	11.1 (3.7), 17	10.8 (4.5), 24	11.4 (4.8), 20	-0.9 (-3.1 to 1.3)	0.9 (-1.4 to 3.1)
reduced motivation (4-20)				Estimated mean difference (95% CI)	
Baseline	14.3 (3.3), 26	12.8 (3.8), 24	11.8 (3.1), 26	-	-
Week 12	10.6 (3.8), 19	10.6 (3.8), 19	10.8 (3.9), 23	0.5 (-1.8 to 2.8)	0.8 (-1.5 to 3.1)
Week 20	10.5 (4.3), 16	10.4 (4.3), 24	10.7 (5.0), 22	0.1 (-2.3 to 2.4)	1.0 (-1.4 to 3.4)
mental fatigue (4-20)				Estimated mean difference (95% CI)	
Baseline	13.3 (3.7), 27	15.5 (4.0), 27	14.0 (4.0), 26	-	-
Week 12	11.6 (4.4), 21	11.6 (4.4), 21	11.9 (4.5), 24	-0.7 (-3.0 to 1.5)	0.0 (-2.2 to 2.1)
Week 20	11.8 (4.4), 18	12.3 (4.4), 25	12.0 (4.3), 22	-0.6 (-2.9 to 1.6)	-0.2 (-2.5 to 2.1)
FFS: frequency and severity of fatigue score (0-8)				Estimated mean difference (95% CI)	
Baseline	4.9 (2.3), 27	5.7 (1.6), 27	5.2 (1.5), 26	-	-
Week 12	4.1 (1.8), 21	3.4 (2.0), 24	3.6 (2.4), 25	-1.2 (-2.3 to -0.1)*	-0.7 (-1.7 to 0.4)
Week 20	3.9 (2.1), 19	3.9 (2.0), 25	3.9 (1.9), 24	-0.6 (-1.7 to 0.5)	-0.3 (-1.4 to 0.8)
HADS					
HADS Anxiety (0-21)				Estimated mean difference (95% CI)	
Baseline	9.3 (4.5), 27	9.6 (4.3), 27	8.6 (4.0), 26	-	-
Week 12	7.1 (4.8), 21	6.7 (4.0), 24	6.5 (4.4), 25	-0.9 (-3.1 to 1.3)	-0.4 (-2.5 to 1.8)
Week 20	6.6 (4.9), 19	7.4 (4.6), 25	6.3 (4.5), 24	0.0 (-2.2 to 2.3)	-0.1 (-2.3 to 2.1)
HADS Depression (0-21)				Estimated mean difference (95% CI)	
Baseline	8.3 (3.9), 27	8.7 (3.9), 27	7.4 (4.7), 26	-	-
Week 12	6.9 (4.7), 21	5.7 (3.4), 24	5.6 (5.1), 25	-1.6 (-3.6 to 0.4)	-0.7 (-2.7 to 1.2)
Week 20	6.5 (5.0), 19	5.9 (3.7), 25	5.2 (4.4), 24	-1.2 (-3.3 to 0.8)	-0.8 (-2.9 to 1.2)
WSAS score (0-40)				Estimated mean difference (95% CI)	
Baseline	19.0 (9.2), 26	21.7 (8.4), 27	19.9 (8.1), 26	-	-
Week 6	16.8 (11.7), 22	12.6 (8.2), 25	11.4 (7.6), 25	-6.1 (-10.7 to -1.5)*	-5.8 (-10.5 to -1.2)*
Week 12	11.7 (9.5), 20	12.8 (9.6), 24	10.8 (8.5), 25	-2.1 (-6.8 to 2.7)	-2.7 (-7.4 to 2.0)
Week 20	13.1 (11.8) 19	16.4 (9.5), 24	12.9 (9.7), 23	0.5 (-4.3 to 5.3)	-1.7 (-6.6 to 3.1)
Week 52	9.8 (10.3), 16	14.3 (9.6), 22	13.1 (10.0), 20	1.2 (-3.8 to 6.3)	1.9 (-3.2 to 7.1)
GSII score (0-10)				Estimated mean difference (95% CI)	
Baseline	3.0 (3.1), 25	2.7 (2.8), 24	3.0 (1.7), 25	-	-
Week 6	4.2 (3.3), 21	6.0 (2.8), 22	5.1 (2.5), 24	1.9 (0.3 to 3.4)*	0.8 (-0.8 to 2.3)
Week 12	6.0 (3.1), 19	6.0 (2.6), 22	5.3 (2.6), 21	0.2 (-1.4 to 1.8)	-0.7 (-2.3 to 0.9)
Week 20	5.8 (3.2), 18	5.0 (2.5), 21	5.1 (3.1), 18	-0.5 (-2.1 to 1.2)	-0.6 (-2.3 to 1.1)
Week 52	6.1 (3.1), 13	5.5 (3.1), 22	5.1 (2.5), 18	-0.4 (-2.2 to 1.4)	-0.9 (-2.7 to 0.9)

SD: standard deviation. Vs: versus. MFI: Multidimensional Fatigue Inventory (35), recall period adapted to the past 2 weeks, subscales 4-20 points, lower score favourable, MCID 2 points for each subscale (34), assessed at baseline, week 12 and 20. CI: confidence interval. FSS: items 4 and 6 of the Flinders Fatigue Scale (36) were added up into frequency and severity of fatigue score, scale 0-8, lower score favourable, assessed at baseline, week 12 and 20. HADS: Hospital Anxiety and Depression Scale (38, 39), recall period adapted to the past 2 weeks, Anxiety and Depression symptoms subscales 0-21, lower score favourable, MCID 1.7 points for each subscale(37), assessed at baseline, week 12 and 20. WSAS: Work and Social Adjustment Scale (40), recall period adapted to the past 2 weeks, scale 0-40, lower score favourable, MCID 8 points (41), assessed at all time points. GSII: Glasgow Sleep Impact Index part 1, 2, and 3 (42), score for rank 1 main personal impairment due to sleep problem scale 0-10, higher score favourable, assessed at all time points.

** p <0.05*

For the continuous outcomes linear mixed model analyses were performed. Models included all measured values from all available time points.

Table S4. Secondary sleep outcomes in the per protocol group per treatment group, comparison between low dose mirtazapine and amitriptyline respectively with placebo. Values are mean score (SD), number, unless stated otherwise.

Outcome measure at each measurement time point	Placebo	Mirtazapine	Amitriptyline	Mirtazapine vs Placebo	Amitriptyline vs placebo
Per protocol analysis	N=15	N=15	N=20		
PSQI					
TST (hours)				Estimated mean difference (95% CI)	
Baseline	4.6 (1.1), 14	4.9 (1.1), 14	4.8 (1.0), 20	-	-
Week 6	5.6 (1.1), 12	6.8 (0.9), 15	6.0 (1.5), 20	1.0 (0.2 to 1.9)*	0.2 (-0.6 to 1.0)
Week 12	5.3 (2.0), 15	7.1 (0.7), 14	6.3 (1.3), 20	1.7 (0.9 to 2.5)*	0.9 (0.2 to 1.7)*
Week 20	5.3 (1.8), 15	6.1 (1.3), 15	5.7 (1.4), 20	0.8 (0.0 to 1.6)	0.5 (-0.3 to 1.2)
Week 52	5.3 (2.2), 10	6.4 (1.5), 14	6.0 (1.4), 19	1.0 (0.1 to 1.9)*	0.6 (-0.2 to 1.5)
SOL (minutes)				Estimated mean difference (95% CI)	
Baseline	48.5 (49.4), 15	35.9 (36.7), 14	60.5 (39.2), 20	-	-
Week 6	37.5 (22.2), 15	22.3 (14.9), 15	81.0 (102.7), 20	-11.2 (-47.4 to 25.0)	39.9 (6.0 to 73.7)*
Week 12	46.8 (44.4), 14	31.4 (30.5), 14	60.8 (60.0), 20	-13.6 (-50.9 to 23.7)	8.9 (-25.6 to 43.3)
Week 20	40.0 (26.9), 15	53.3 (60.4), 15	48.8 (40.9), 20	17.2 (-19.0 to 53.4)	5.1 (-28.7 to 38.9)
Week 52	69.5 (103.5), 10	36.4 (38.0), 14	45.1 (57.2), 19	-32.5 (-73.0 to 8.0)	-28.9 (-67.1 to 9.3)
SE (%)				Estimated mean difference (95% CI)	
Baseline	55.9 (16.2), 13	58.6 (12.6), 14	55.7 (9.5), 20	-	-
Week 6	65.4 (15.3), 12	79.9 (12.4), 15	65.2 (15.5), 20	9.9 (-0.5 to 20.2)	-2.1 (-11.8 to 7.6)
Week 12	71.1 (30.4), 14	81.8 (9.5), 14	69.0 (19.2), 20	12.7 (2.6 to 22.8)*	1.3 (-8.0 to 10.7)
Week 20	69.3 (19.8), 14	71.0 (15.8), 14	66.4 (15.3), 20	2.1 (-8.1 to 12.2)	0.5 (-8.8 to 9.9)
Week 52	62.1 (26.5), 10	77.4 (17.3), 14	68.6 (15.5), 19	12.7 (1.8 to 23.6)*	6.2 (-4.0 to 16.4)
Frequency of sleep problem; 3 or more days per week (No(%), n)				OR (95% CI)	
Baseline	13 (86.7), 15	13 (86.7), 15	20 (100.0), 20	-	-
Week 6	10 (66.7), 15	3 (20.0), 15	12 (60.0), 20	0.1 (0.0 to 0.5)*	0.4 (0.1 to 2.0)
Week 12	8 (53.3), 15	2 (13.3), 15	11 (55.5), 20	0.1 (0.0 to 0.8)*	0.9 (0.2 to 3.7)
Week 20	9 (60.0), 15	9 (60.0), 15	11 (55.0), 20	0.7 (0.2 to 3.2)	0.5 (0.1 to 2.1)
Week 52	8 (72.7), 11	5 (35.7), 14	12 (63.2), 19	0.2 (0.0 to 1.1)	0.5 (0.1 to 2.9)

**Table S4.** Continued					
Consensus Sleep Diary					
TST (hours)				Estimated mean difference (95% CI)	
Baseline<br>Week 6<br>Week 20	5.3 (1.1), 15<br>5.8 (1.3), 15<br>5.7 (1.7), 14	5.0 (1.1), 13<br>7.2 (0.9), 13<br>6.1 (1.4), 13	5.6 (1.0), 18<br>6.6 (1.4), 16<br>6.5 (1.5), 16	-<br>1.6 (0.9 to 2.3)*<br>0.6 (-0.2 to 1.3)	-<br>0.5 (-0.2 to 1.1)<br>0.5 (-0.2 to 1.1)
SOL (minutes)				Estimated mean difference (95% CI)	
Baseline<br>Week 6<br>Week 20	74.9 (48.0), 15<br>70.6 (59.9), 15<br>81.7 (116.5), 14	61.1 (47.9), 13<br>31.6 (28.8), 13<br>54.2 (44.5), 13	49.1 (29.3), 18<br>65.9 (66.3), 16<br>43.7 (44.7), 16	-<br>-33.0 (-71.7 to 5.7)<br>-21.6 (-60.9 to 17.6)	-<br>8.8 (-27.8 to 45.4)<br>-25.0 (-62.2 to 12.3)
SE (TST/TIB*100%)				Estimated mean difference (95% CI)	
Baseline<br>Week 6<br>Week 20	61.5 (11.9), 15<br>66.3 (17.1), 15<br>64.7 (14.9), 14	59.6 (13.0), 13<br>79.4 (8.1), 13<br>68.9 (12.4), 13	66.2 (11.7), 18<br>73.5 (14.3), 16<br>73.9 (15.8), 16	-<br>14.3 (6.5 to 22.1)*<br>4.9 (-3.1 to 12.8)	-<br>2.8 (-4.6 to 10.2)<br>4.6 (-2.9 to 12.1)
WASO (minutes)				Estimated mean difference (95% CI)	
Baseline<br>Week 6<br>Week 20	128.3 (57.1), 15<br>114.1 (52.6), 15<br>105.3 (52.0), 14	142.3 (42.8), 13<br>79.3 (37.9), 13<br>106.0 (64.8), 13	125.7 (59.2), 18<br>81.2 (45.5), 16<br>97.1 (56.1), 16	-<br>-41.6 (-75.1 to -8.1)*<br>-3.6 (-37.5 to 30.4)	-<br>-28.4 (-60.1 to 3.2)<br>-0.5 (-32.7 to 31.8)
NWAK				Estimated mean difference (95% CI)	
Baseline<br>Week 6<br>Week 20	1.3 (0.7), 15<br>1.4 (0.7), 15<br>1.4 (0.8), 14	1.2 (0.5), 13<br>1.2 (0.6), 13<br>1.2 (0.7), 13	1.4 (0.6), 18<br>1.0 (0.8), 16<br>1.0 (0.9), 16	-<br>-0.1 (-0.6 to 0.3)<br>-0.2 (-0.6 to 0.3)	-<br>-0.3 (-0.8 to 0.1)<br>-0.4 (-0.8 to 0.0)
TWAK (minutes)				Estimated mean difference (95% CI)	
Baseline<br>Week 6<br>Week 20	63.2 (54.5), 15<br>41.3 (35.7), 15<br>39.5 (35.4), 14	66.6 (42.2), 13<br>36.9 (25.2), 13<br>40.7 (30.2), 13	46.7 (34.9), 18<br>41.0 (27.0), 16<br>47.8 (42.7), 16	-<br>-5.8 (-30.8 to 19.3)<br>0.9 (-24.6 to 26.3)	-<br>7.5 (-16.3 to 31.2)<br>17.3 (-6.9 to 41.4)
Sleep Quality (1-10)				Estimated mean difference (95% CI)	
Baseline<br>Week 6<br>Week 20	5.1 (1.3), 11<br>6.4 (1.3), 12<br>5.4 (1.9), 11	4.4 (0.8), 13<br>6.9 (1.0), 12<br>5.2 (1.2), 11	5.1 (0.8), 18<br>6.4 (1.2), 14<br>6.0 (1.8), 13	-<br>0.9 (0.0 to 1.8)<br>0.1 (-0.8 to 1.0)	-<br>0.1 (-0.8 to 0.9)<br>0.6 (-0.3 to 1.5)
Refreshing Quality of Sleep (1-10)				Estimated mean difference (95% CI)	
Baseline<br>Week 6<br>Week 20	5.1 (1.8), 10<br>6.1 (1.3), 15<br>5.9 (1.9), 11	4.5 (1.0), 13<br>6.5 (0.7), 12<br>5.4 (1.0), 11	5.2 (1.0), 18<br>6.3 (1.1), 14<br>5.9 (1.7), 13	-<br>0.5 (-0.5 to 1.4)<br>-0.3 (-1.3 to 0.7)	-<br>0.1 (-0.8 to 1.0)<br>0.1 (-0.8 to 1.1)

**Table S4.** Continued					
Global rate of change (-5 to 5)				Estimated mean difference (95% CI)	
Week 6	0.6 (2.5), 15	3.0 (1.3), 15	2.0 (1.3), 20	2.4 (1.0 to 3.8)*	1.4 (0.0 to 2.7)*
Week 12	0.3 (2.8), 14	3.1 (1.2), 15	2.3 (1.9), 20	2.8 (1.4 to 4.2)*	2.0 (0.7 to 3.3)*
Week 20	0.5 (2.4), 14	1.8 (1.6), 15	1.1 (2.4), 19	1.4 (0.0 to 2.8)	0.6 (-0.7 to 2.0)

*SD: standard deviation. Vs: versus. PSQI: Pittsburgh Sleep Quality Index (31), item 1-4, recall period adapted to the past 2 weeks, assessed at all time points. TST: Total Sleep Time, hours. CI: confidence interval. SOL: Sleep Onset Latency, minutes, number of minutes it takes to fall asleep. SE: sleep efficiency, 0-100%, total sleep time divided by time between lights out time and final rise time *100%. Frequency of sleep problem: reported number of nights per week experiencing sleep problem, % reporting on 3 or more nights per week, assessed at all time points. Consensus sleep diary(32): prospectively filled out over one week at baseline, week6 and 20. TIB: time in and out bed, hours, time between lights out time and final rise time. WASO: Wake time After Sleep Onset, minutes, total minutes awake after sleep onset and before final rise time regardless whether it was spend in or out bed. NWAK: Number of awakenings, number of night-time awakenings per night. TWAK: terminal wakefulness, minutes, time between final awakening and final rise time. Sleep Quality: sleep quality score, scale 1-10, higher score favourable. Refreshing Quality of Sleep: refreshing Quality of Sleep score, scale 1-10, higher score favourable. Global rate of change (GRC) (33): scale -5 to 5, assessing to what extent the sleep problem was experienced as being improved, worsened or unchanged compared to baseline; higher score favourable, MCID 2 points, assessed at week 6, 12 and 20.*

** p <0.05*

For the continuous outcomes linear mixed model analyses were performed. For dichotomous outcomes, logistic generalized estimating equations (GEE) analysis was used. Models included all measured values from all available time points.

Table S5. Secondary daytime outcomes in the per protocol group per treatment group, comparison between low dose mirtazapine and amitriptyline respectively with placebo. Values are mean score (SD), number, unless stated otherwise

Outcome measure at each measurement time point	Placebo	Mirtazapine	Amitriptyline	Mirtazapine vs Placebo	Amitriptyline vs placebo
Per protocol analysis	N=15	N=15	N=20		
MFI					
general fatigue (4-20)				Estimated mean difference (95% CI)	
Baseline<br>Week 12<br>Week 20	16.4 (4.2), 15<br>13.5 (3.2), 14<br>12.5 (3.9), 14	18.2 (2.7), 15<br>11.1 (4.5), 14<br>12.9 (4.3), 15	16.8 (2.0), 20<br>14.1 (4.4), 19<br>14.8 (4.3), 17	-<br>-2.9 (-5.5 to -0.3)*<br>-0.2 (-2.7 to 2.4)	-<br>0.5 (-2.0 to 2.9)<br>2.1 (-0.3 to 4.6)
physical fatigue (4-20)				Estimated mean difference (95% CI)	
Baseline<br>Week 12<br>Week 20	14.0 (3.5), 15<br>12.4 (3.9), 14<br>11.5 (3.3), 14	14.7 (4.7), 15<br>9.8 (3.9), 14<br>10.9 (4.1), 15	14.5 (2.8), 20<br>12.5 (4.2), 18<br>14.1 (4.3), 15	-<br>-2.7 (-5.0 to -0.4)*<br>-0.9 (-3.2 to 1.3)	-<br>-0.1 (-2.2 to 2.1)<br>2.5 (0.2 to 4.7)
reduced activity (4-20)				Estimated mean difference (95% CI)	
Baseline<br>Week 12<br>Week 20	14.3 (4.0), 15<br>12.3 (4.2), 14<br>11.6 (3.3), 13	14.6 (4.5), 13<br>9.1 (2.9), 12<br>11.1 (4.6), 15	13.4 (3.5), 19<br>12.2 (4.3), 18<br>11.5 (4.8), 17	-<br>-3.2 (-5.9 to -0.6)*<br>-0.8 (-3.4 to 1.8)	-<br>0.3 (-2.1 to 2.7)<br>0.5 (-2.0 to 2.9)
reduced motivation (4-20)				Estimated mean difference (95% CI)	
Baseline<br>Week 12<br>Week 20	13.8 (3.6), 15<br>11.0 (3.7), 14<br>11.1 (3.8), 13	12.7 (3.6), 13<br>10.3 (4.7), 13<br>10.3 (4.8), 15	11.9 (2.8), 20<br>10.7 (3.7), 18<br>10.8 (5.1), 18	-<br>-0.5 (-3.4 to 2.3)<br>-0.6 (-3.4 to 2.2)	-<br>0.4 (-2.2 to 3.0)<br>0.3 (-2.4 to 3.0)
mental fatigue (4-20)				Estimated mean difference (95% CI)	
Baseline<br>Week 12<br>Week 20	12.1 (3.8), 15<br>11.9 (4.6), 15<br>12.3 (4.5), 14	15.7 (3.9), 15<br>10.1 (4.1), 14<br>12.1 (4.5), 15	14.5 (3.3), 20<br>11.8 (4.4), 19<br>12.2 (4.3), 18	-<br>-3.2 (-5.9 to -0.5)*<br>-1.8 (-4.5 to 0.9)	-<br>-1.1 (-3.6 to 1.4)<br>-1.1 (-3.6 to 1.5)
FFS: Frequency and severity of fatigue score (0-8)				Estimated mean difference (95% CI)	
Baseline<br>Week 12<br>Week 20	4.3 (2.5), 15<br>4.5 (1.7), 15<br>4.1 (2.3), 15	6.0 (1.5), 15<br>3.2 (1.9), 15<br>3.8 (2.0), 15	5.4 (1.5), 20<br>3.9 (2.4), 20<br>4.2 (1.8), 20	-<br>-2.0 (-3.3 to -0.7)*<br>-1.1 (-2.4 to 0.2)	-<br>-1.1 (-2.3 to 0.1)<br>-0.4 (-1.6 to 0.8)
HADS					
HADS Anxiety (0-21)				Estimated mean difference (95% CI)	
Baseline<br>Week 12<br>Week 20	7.3 (4.1), 15<br>7.2 (4.6), 15<br>6.8 (5.5), 15	10.7 (4.1), 15<br>6.5 (4.4), 15<br>7.7 (4.9), 15	8.3 (4.1), 20<br>6.4 (4.2), 20<br>6.7 (4.7), 20	-<br>-2.8 (-5.4 to -0.2)*<br>-1.4 (-3.9 to 1.0)	-<br>-1.2 (-3.8 to 1.4)<br>-0.7 (-3.2 to 1.7)
HADS Depression (0-21)				Estimated mean difference (95% CI)	
Baseline<br>Week 12<br>Week 20	7.9 (4.4), 15<br>7.7 (4.2), 15<br>7.7 (4.7), 15	9.7 (4.4), 15<br>5.3 (3.7), 15<br>6.2 (4.3), 15	7.1 (4.7), 20<br>5.3 (5.2), 20<br>5.7 (4.6), 20	-<br>-3.6 (-6.1 to -1.2)*<br>-1.9 (-4.2 to 0.4)	-<br>-2.7 (-5.1 to -0.3)*<br>-1.5 (-3.8 to 0.7)
WSAS score (0-40)				Estimated mean difference (95% CI)	
Baseline<br>Week 6<br>Week 12<br>Week 20<br>Week 52	16.9 (9.8), 14<br>14.3 (11.0), 15<br>11.9 (9.7), 14<br>15.3 (12.2), 15<br>9.2 (11.8), 11	25.0 (6.2), 15<br>12.2 (9.2), 15<br>10.9 (9.1), 15<br>17.0 (10.0), 14<br>12.3 (9.1), 14	19.9 (7.5), 20<br>11.3 (7.3), 20<br>10.5 (8.0), 20<br>12.8 (9.8), 20<br>13.7 (10.0), 18	-<br>-6.3 (-12.0 to -0.6)*<br>-6.6 (-12.4 to -0.8)*<br>-2.3 (-8.1 to 3.5)<br>-1.7 (-7.9 to 4.5)	-<br>-4.5 (-9.9 to 0.8)<br>-4.3 (-9.7 to 1.2)<br>-4.0 (-9.3 to 1.4)<br>2.1 (-3.7to 8.0)
GSII score (0-10)				Estimated mean difference (95% CI)	
Baseline<br>Week 6<br>Week 12<br>Week 20<br>Week 52	2.3 (2.9), 15<br>4.5 (3.5), 15<br>5.8 (3.2), 14<br>4.9 (3.0), 14<br>6.4 (3.2), 9	2.4 (2.5), 14<br>6.8 (2.7), 12<br>7.0 (2.4), 14<br>5.1 (2.3), 13<br>6.4 (3.1), 14	2.9 (1.7), 19<br>5.2 (2.6), 19<br>5.4 (2.6), 17<br>5.4 (3.2), 16<br>5.4 (2.5), 15	-<br>2.2 (0.3 to 4.1)*<br>1.3 (-0.6 to 3.1)<br>0.2 (-1.6 to 2.1)<br>0.0 (-2.1 to 2.1)	-<br>0.5 (-1.2 to 2.2)<br>-0.7 (-2.5 to 1.0)<br>0.1 (-1.7 to 1.9)<br>-1.3 (-3.3 to 0.8)

SD: standard deviation. Vs: versus. MFI: Multidimensional Fatigue Inventory (35), recall period adapted to the past 2 weeks, subscales 4-20 points, lower score favourable, MCID 2 points for each subscale (34), assessed at baseline, week 12 and 20. CI: confidence interval. FSS: items 4 and 6 of the Flinders Fatigue Scale (36) were added up into frequency and severity of fatigue score, scale 0-8, lower score favourable, assessed at baseline, week 12 and 20. HADS: Hospital Anxiety and Depression Scale (38, 39), recall period adapted to the past 2 weeks, Anxiety and Depression symptoms subscales 0-21, lower score favourable, MCID 1.7 points for each subscale(37), assessed at baseline, week 12 and 20. WSAS: Work and Social Adjustment Scale (40), recall period adapted to the past 2 weeks, scale 0-40, lower score favourable, MCID 8 points (41), assessed at all time points. GSII: Glasgow Sleep Impact Index part 1, 2, and 3 (42), score for rank 1 main personal impairment due to sleep problem scale 0-10, higher score favourable, assessed at all time points.

** $p < 0.05$*

For the continuous outcomes linear mixed model analyses were performed. Models included all measured values from all available time points.

Table S6. Post hoc analysis comparison of primary outcome (ISI score) between treatment groups and placebo, crude analysis, and analysis adjusted for sex and analysis adjusted for comorbidity.

Outcome measure	Mirtazapine vs Placebo	Amitriptyline vs Placebo	Mirtazapine vs Placebo	Amitriptyline vs Placebo	Mirtazapine vs Placebo	Amitriptyline vs Placebo
Intention to treat	Crude analysis		Adjusted for sex		Adjusted for comorbidity	
ISI score (0-28)	Estimated mean difference (95% CI)		Estimated mean difference (95% CI)		Estimated mean difference (95% CI)	
Baseline	-	-	-	-	-	-
Week 6	-6.0 (-9.0 to -3.0)*	-3.4 (-6.3 to -0.4)*	-6.0 (-8.9 to -3.0)*	-3.3 (-6.3 to -0.4)*	-6.0 (-9.0 to -3.1)*	-3.5 (-6.4 to -0.5)*
Week 12	-1.9 (-5.0 to 1.1)	-0.6 (-3.6 to 2.4)	-1.9 (-4.9 to 1.1)	-0.6 (-3.6 to 2.4)	-2.0 (-5.0 to 1.0)	-0.7 (-3.7 to 2.3)
Week 20	0.7 (-2.3 to 3.8)	0.2 (-2.9 to 3.2)	0.8 (-2.3 to 3.8)	0.2 (-2.9 to 3.3)	0.7 (-2.4 to 3.7)	0.1 (-3.0 to 3.1)
Week 52	-1.2 (-4.5 to 2.1)	1.8 (-1.5 to 5.0)	-1.2 (-4.4 to 2.1)	1.8 (-1.5 to 5.1)	-1.2 (-4.5 to 2.0)	1.7 (-1.6 to 4.9)

ISI: Insomnia Severity Index(26, 27), recall period adapted to the past 2 weeks. Vs: versus. CI: confidence interval

** p <0.05*

For the continuous outcomes linear mixed model analyses were performed. Models included all measured values from all available time points.

Table S7. Treatment tolerability in the intention-to-treat group or in the per protocol group per treatment group, comparison between low-dose mirtazapine, and amitriptyline respectively with placebo. Values are mean score (SD), number, unless stated otherwise

Outcome measure at each measurement time point	Placebo	Mirtazapine	Amitriptyline	Mirtazapine vs Placebo	Amitriptyline vs placebo
Intention to treat analysis	N=27	N=27	N =26		
ASEC					
Mean (SD) count of ASEC health complaints				Poisson rate ratio (95% CI)	
Baseline<br>Week 6†<br>Week 12‡	7.7 (6.3), 27<br>6.7 (6.7), 21<br>6.2 (6.8), 18	7.6 (4.9), 27<br>4.8 (3.8), 25<br>5.0 (3.2), 21	5.4 (2.8), 26<br>3.3 (1.9), 24<br>3.8 (2.7), 24	1.1 (0.8 to 1.6)<br>0.8 (0.6 to 1.3)<br>0.9 (0.6 to 1.4)	0.8 (0.6 to 1.2)<br>0.6 (0.4 to 0.9)*<br>0.8 (0.5 to 1.2)
Mean (SD) count of ASEC side effects				Poisson rate ratio (95% CI)	
Week 6†<br>Week 12‡	1.8 (3.4), 21<br>0.9 (1.4), 18	3.6 (3.9), 25<br>2.8 (2.8), 21	1.8 (1.8), 24<br>2.3 (2.7), 24	2.2 (1.1 to 4.4)*<br>3.5 (1.5 to 8.1)*	1.1 (0.5 to 2.3)<br>2.9 (1.3 to 6.8)*
≥1 ASEC side effect§ (No (%),n)				Odds ratio (95% CI)	
Week 6†<br>Week 12‡	10 (47.6), 21<br>7 (38.9), 18	21 (84.0), 25<br>15 (71.4), 21	16 (66.7), 24<br>14 (58.3), 24	5.8 (1.5 to 22.7)*<br>3.5 (0.9 to 12.6)	2.2 (0.7 to 7.3)<br>2.1 (0.6 to 7.0)
Self-reported bodyweight					
Mean (SD) weight (kg)<br>Intention to treat group	N=27	N=27	N =26	Estimated mean difference (95% CI)	
Baseline<br>Week 6<br>Week 12<br>Week 20<br>Week 52	73.7 (15.5), 27<br>76.0 (16.4), 22<br>76.3 (17.1), 21<br>74.4 (15.4), 19<br>78.2 (18.1), 16	67.8 (10.1), 27<br>68.3 (10.4), 25<br>68.6 (10.6), 24<br>68.5 (11.0), 24<br>66.9 (9.9), 22	69.7 (12.0), 25<br>72.0 (12.9), 24<br>72.3 (13.0), 25<br>71.2 (13.3), 24<br>70.3 (13.8), 22	-<br>0.7 (-0.9 to 2.3)<br>0.9 (-0.7 to 2.5)<br>0.4 (-1.3 to 2.0)<br>-0.7 (-2.5 to 1.0)	-<br>0.5 (-1.1 to 2.1)<br>0.6 (-1.0 to 2.3)<br>-0.7 (-2.3 to 1.0)<br>-1.7 (-3.4 to 0.0)
Mean (SD) weight (kg)<br>Per protocol group¶	N=15	N=15	N=20	Estimated mean difference (95% CI)	
Baseline<br>Week 6<br>Week 12<br>Week 20<br>Week 52	76.6 (15.5), 15<br>77.1 (15.6), 15<br>77.6 (16.1), 15<br>77.8 (15.6), 15<br>79.3 (17.7), 11	64.4 (7.6), 15<br>66.3 (7.9), 15<br>66.4 (8.3), 15<br>66.2 (8.5), 15<br>65.1 (8.9), 14	70.2 (11.7), 19<br>72.7 (12.3), 20<br>72.9 (12.8), 20<br>72.1 (13.2), 20<br>71.3 (13.3), 19	-<br>1.1 (-0.8 to 3.1)<br>0.8 (-1.2 to 2.8)<br>0.4 (-1.6 to 2.4)<br>-0.6 (-2.7 to 1.5)	-<br>0.7 (-1.1 to 2.6)<br>0.5 (-1.4 to 2.3)<br>-0.5 (-2.4 to 1.3)<br>-0.8 (-2.8 to 1.2)
Treatment discontinuation rate, due to side-effects** (cumulative No (%), n)	N=25	N=27	N=26	Odds ratio (95% CI)††	
Week 6<br>Week 12<br>16 week treatment	1 (4.0), 25<br>2 (8.0), 25<br>2 (8.0), 25	4 (14.8), 27<br>7 (25.9), 27<br>8 (29.6), 27	1 (3.8), 26<br>1 (3.8), 26<br>1 (3.8), 26	4.2 (0.4 to 40.2)<br>4.0 (0.7 to 21.6)<br>4.8 (0.9 to 25.6)	1.0 (0.1 to 16.2)<br>0.5 (0.0 to 5.4)<br>0.5 (0.4 to 5.4)
Tolerability of side-effects	N=25	N=27	N=26		
Did you find the possible side effects of the DREAMING treatment tablets tolerable/acceptable?<br>I had no side effects<br>Very tolerable/acceptable<br>Somewhat tolerable/acceptable<br>Not tolerable/acceptable<br>Missing	 10 (40.0) (50.0)<br>9 (36.0) (45.0)<br>1 (4.0) (5.0)<br>0 (0.0) (0.0)<br>5 (20.0)	 4 (14.8) (16.0)<br>7 (25.9) (28.0)<br>9 (33.3) (36.0)<br>5 (18.5) (20.0)<br>2 (7.4)	 7 (26.9) (29.2)<br>12 (46.2) (50.0)<br>2 (7.7) (8.3)<br>3 (11.5)(12.5)<br>2 (7.7)		
DESS					
Mean (SD) count of DESS discontinuation symptoms				Poisson rate ratio (95% CI)	
20 weeks / 4 weeks after stop	2.0 (2.9), 20	1.2 (2.0), 25	2.4 (3.7), 24	0.6 (0.4 to 1.0)*	1.2 (0.8 to 1.8)
≥4 DESS symptoms (No (%), n)				OR (95% CI)	
20 weeks / 4 weeks after stop	4 (20.0), 20	4 (16.0)	5 (20.8)	0.8 (0.2 to 3.5)	1.1 (0.2 to 4.6)

Table S7. continued

Rebound insomnia reported(No(total%)(valid%)) Per protocol group¶	N=15	N=15	N=20		
In the first week after you stopped taking the DREAMING treatment tablets, did you notice that your sleep problem came back (temporarily) or got worse?					
No, in the week after stopping, my sleep was similar or better	8 (53.3)	6 (40.0)	7 (35.0)(38.9)		
Yes, in the week after stopping I slept temporarily worse again	0 (0.0)	3 (20.0)	4 (20.0)(22.2)		
Yes, since stopping I sleep worse again	7 (46.7)	6 (40.0)	7 (35.0)(38.9)		
Missing	0 (0.0)	0 (0.0)	2 (10.0)		

SD: standard deviation. Vs: versus. ASEC: Antidepressant Side-Effect Checklist (43, 44), recall period adapted to past 2 weeks, the items 'difficulty sleeping' and 'drowsiness' in the ASEC-21 were replaced by the items 'difficulty waking up', 'drowsiness in the morning', 'drowsiness in the afternoon', 'restless sleep' and 'vivid dreams, assessed at baseline, week 6 and 12 and in case of treatment discontinuation as soon as possible. Health complaints: all reported ASEC health complaints over the past 2 weeks. Side effects: ASEC health complaints attributed to the treatment by the participant. CI: confidence interval. Self-reported body weight, patients were instructed to weight themselves with clothes, but without shoes, assessed at all time points. Tolerability of side-effects, assessed at week 20 and in case of treatment discontinuation in combination with the next planned questionnaire. DESS: Discontinuation-Emergent Signs and Symptoms (45, 46); retrospective over the week after stop, the item 'sleep problem' was omitted from the DESS list and (rebound) insomnia was evaluated in a separate item, assessed at week 20 and in case of treatment discontinuation with the next planned questionnaire.

** p <0.05. †Week 6: week 1-6; the ASEC questionnaire was sent to those participant who were on treatment at week 6 or as soon as possible after discontinuation before week 6. 78 participants started treatment; missings on this questionnaire were 4, 2, 2 in the treatment groups respectively. ‡Week 12: week 7-12; the ASEC questionnaire was sent to those participants who were on treatment at week 12 or as soon as possible after discontinuation after week 6 and before week 12. 68 participants were on treatment after week 6; missings on this questionnaire were 4, 1, 0 in the treatment groups respectively. § Number of patients reporting at least one side effect, irrespectively of severity. At least one severe side effect was reported at week 6 by 40.0, 25.0, and 14.3% in the treatment groups respectively and at week 12 by 33.3, 29.2, and 0.0% in the treatment groups respectively. ¶ Per protocol: on treatment 16 weeks and reported no or less than 10 days of missed tablets.** Reasons for treatment discontinuation were assessed by the researchers by phone call. The provided main reasons for discontinuations in the mirtazapine group were drowsiness (5x), weight gain (2x), restlessness (1x). The type of side-effects leading to discontinuation in the other groups was unspecified.††Odds ratios were independently calculated for each time point. For the continuous outcomes linear mixed model analyses were performed. For dichotomous outcomes, logistic generalized estimating equations (GEE) analysis was used. For count data, Poisson mixed model regression analysis was performed. Models included all measured values from all available time points, unless otherwise specified.*

Table S8. Number of participants reporting with a particular side effects (i.e. ASEC health complaints attributed to treatment) at 6 weeks, total and categorized by treatment and severity of symptoms (mild/moderate/severe) Values are numbers (column percentages) unless stated otherwise.

	Placebo N=21				Mirtazapine N=25				Amitriptyline N=24			
ASEC-21	Total	Mild	Moderate	Severe	Total	Mild	Moderate	Severe	Total	Mild	Moderate	Severe
Dry mouth	4 (19.0)	1 (4.8)	3 (14.3)	0 (0.0)	6 (24.0)	1 (4.0)	4 (16.0)	1 (4.0)	7 (29.2)	0 (0.0)	6 (25.0)	1 (4.2)
Difficulty of waking up	1 (4.8)	0 (0.0)	1 (4.8)	0 (0.0)	7 (28.0)	3 (12.0)	2 (8.0)	2 (8.0)	4 (16.7)	1 (4.2)	2 (8.3)	1 (4.2)
Drowsiness in the morning	3 (14.3)	1 (4.8)	1 (4.8)	1 (4.8)	14 (56.0)	2 (8.0)	8 (32.0)	4 (16.0)	8 (33.3)	2 (8.3)	4 (16.7)	2 (8.3)
Drowsiness in the afternoon	4 (19.0)	0 (0.0)	3 (14.3)	1 (4.8)	6 (24.0)	1 (4.0)	3 (12.0)	2 (8.0)	4 (16.7)	4 (16.7)	0 (0.0)	0 (0.0)
Restless sleep	1 (4.8)	0 (0.0)	0 (0.0)	1 (4.8)	4 (16.0)	2 (8.0)	1 (4.0)	1 (4.0)	2 (8.3)	1 (4.2)	0 (0.0)	1 (4.2)
Vivid dreams	2 (9.5)	0 (0.0)	0 (0.0)	2 (9.5)	9 (36.0)	0 (0.0)	7 (28.0)	2 (8.0)	7 (29.2)	2 (8.3)	2 (8.3)	3 (12.5)
Blurred vision	2 (9.5)	1 (4.8)	0 (0.0)	1 (4.8)	2 (8.0)	0 (0.0)	2 (8.0)	0 (0.0)	3 (12.5)	1 (4.2)	2 (8.3)	0 (0.0)
Headache	3 (14.3)	1 (4.8)	2 (9.5)	0 (0.0)	4 (16.0)	2 (8.0)	1 (4.0)	1 (4.0)	0 (0.0)	0 (0.0)	0 (0.0)	0 (0.0)
Constipation	0 (0.0)	0 (0.0)	0 (0.0)	0 (0.0)	6 (24.0)	2 (8.0)	2 (8.0)	2 (8.0)	1 (4.2)	0 (0.0)	0 (0.0)	1 (4.2)
Diarrhea	0 (0.0)	0 (0.0)	0 (0.0)	0 (0.0)	1 (4.0)	1 (4.0)	0 (0.0)	0 (0.0)	0 (0.0)	0 (0.0)	0 (0.0)	0 (0.0)
Appetite increase	2 (9.5)	0 (0.0)	1 (4.8)	1 (4.8)	7 (28.0)	0 (0.0)	4 (16.0)	3 (12.0)	3 (12.5)	1 (4.2)	2 (8.3)	0 (0.0)
Appetite decrease	0 (0.0)	0 (0.0)	0 (0.0)	0 (0.0)	0 (0.0)	0 (0.0)	0 (0.0)	0 (0.0)	0 (0.0)	0 (0.0)	0 (0.0)	0 (0.0)
Nausea, vomiting	0 (0.0)	0 (0.0)	0 (0.0)	0 (0.0)	0 (0.0)	0 (0.0)	0 (0.0)	0 (0.0)	0 (0.0)	0 (0.0)	0 (0.0)	0 (0.0)
Urination problems	0 (0.0)	0 (0.0)	0 (0.0)	0 (0.0)	0 (0.0)	0 (0.0)	0 (0.0)	0 (0.0)	0 (0.0)	0 (0.0)	0 (0.0)	0 (0.0)
Sexual problems	0 (0.0)	0 (0.0)	0 (0.0)	0 (0.0)	3 (12.0)	1 (4.0)	1 (4.0)	1 (4.0)	0 (0.0)	0 (0.0)	0 (0.0)	0 (0.0)
Palpitations	1 (4.8)	0 (0.0)	1 (4.8)	0 (0.0)	2 (8.0)	0 (0.0)	1 (4.0)	1 (4.0)	0 (0.0)	0 (0.0)	0 (0.0)	0 (0.0)
Orthostatic hypotension	2 (9.5)	0 (0.0)	0 (0.0)	2 (9.5)	2 (8.0)	1 (4.0)	1 (4.0)	0 (0.0)	1 (4.2)	1 (4.2)	0 (0.0)	0 (0.0)
Vertigo	2 (9.5)	0 (0.0)	1 (4.8)	1 (4.8)	0 (0.0)	0 (0.0)	0 (0.0)	0 (0.0)	0 (0.0)	0 (0.0)	0 (0.0)	0 (0.0)
Sweating	2 (9.5)	0 (0.0)	0 (0.0)	2 (9.5)	2 (8.0)	0 (0.0)	2 (8.0)	0 (0.0)	0 (0.0)	0 (0.0)	0 (0.0)	0 (0.0)
Increased temperature	1 (4.8)	0 (0.0)	1 (4.8)	0 (0.0)	1 (4.0)	0 (0.0)	1 (4.0)	0 (0.0)	0 (0.0)	0 (0.0)	0 (0.0)	0 (0.0)
Tremor	1 (4.8)	0 (0.0)	0 (0.0)	1 (4.8)	1 (4.0)	1 (4.0)	0 (0.0)	0 (0.0)	0 (0.0)	0 (0.0)	0 (0.0)	0 (0.0)
Disorientation	1 (4.8)	0 (0.0)	1 (4.8)	0 (0.0)	3 (12.0)	1 (4.0)	2 (8.0)	0 (0.0)	0 (0.0)	0 (0.0)	0 (0.0)	0 (0.0)
Yawning	2 (9.5)	0 (0.0)	1 (4.8)	1 (4.8)	1 (4.0)	1 (4.0)	0 (0.0)	0 (0.0)	1 (4.2)	0 (0.0)	0 (0.0)	1 (4.2)
Weight gain	3 (14.3)	1 (4.8)	2 (9.5)	0 (0.0)	9 (36.0)	2 (8.0)	3 (12.0)	4 (16.0)	4 (16.7)	1 (4.2)	2 (8.3)	1 (4.2)

ASEC: Antidepressant Side-Effect Checklist(43, 44), Adapted for self-administration in insomnia disorder patients. The items 'difficulty sleeping' and 'drowsiness' in the ASEC-21 were replaced by the items 'difficulty waking up', 'drowsiness in the morning', 'drowsiness in the afternoon', 'restless sleep' and 'vivid dreams'.

This questionnaire was sent to those participants who were on treatment at week 6 or as soon as possible after discontinuation before week 6. 78 participants started treatment; missings on this questionnaire were 4, 2, 2 in the treatment groups respectively.

Table S9. Number of participants reporting with a particular side effects (i.e. ASEC health complaints attributed to treatment) at 12 weeks, total and categorized by treatment and severity of symptoms (mild/moderate/severe) Values are numbers (column percentages) unless stated otherwise.

	Placebo N=18				Mirtazapine N=21				Amitriptyline N=24			
ASEC-21	Total	Mild	Moderate	Severe	Total	Mild	Moderate	Severe	Total	Mild	Moderate	Severe
Dry mouth	1 (5.6)	0 (0.0)	1 (5.6)	0 (0.0)	5 (23.8)	4 (19.0)	0 (0.0)	1 (4.8)	5 (20.8)	1 (4.2)	4 (16.7)	1 (4.2)
Difficulty of waking up	1 (5.6)	1 (5.6)	0 (0.0)	0 (0.0)	8 (38.1)	2 (9.5)	2 (9.5)	4 (19.0)	6 (25.0)	3 (12.5)	3 (12.5)	0 (0.0)
Drowsiness in the morning	2 (11.1)	2 (11.1)	0 (0.0)	0 (0.0)	5 (23.8)	0 (0.0)	3 (14.3)	2 (9.5)	8 (33.3)	3 (12.5)	3 (12.5)	2 (8.3)
Drowsiness in the afternoon	2 (11.1)	1 (5.6)	1 (5.6)	0 (0.0)	3 (14.3)	0 (0.0)	2 (9.5)	1 (4.8)	3 (12.5)	2 (8.3)	1 (4.2)	0 (0.0)
Restless sleep	0 (0.0)	0 (0.0)	0 (0.0)	0 (0.0)	3 (14.3)	2 (9.5)	1 (4.8)	0 (0.0)	4 (16.7)	0 (0.0)	3 (12.5)	1 (4.2)
Vivid dreams	3 (16.7)	2 (11.1)	1 (5.6)	0 (0.0)	8 (38.1)	3 (14.3)	3 (14.3)	2 (9.5)	6 (25.0)	0 (0.0)	3 (12.5)	3 (12.5)
Blurred vision	0 (0.0)	0 (0.0)	0 (0.0)	0 (0.0)	3 (14.3)	2 (9.5)	1 (4.8)	0 (0.0)	1 (4.2)	0 (0.0)	1 (4.2)	0 (0.0)
Headache	1 (5.6)	0 (0.0)	1 (5.6)	0 (0.0)	0 (0.0)	0 (0.0)	0 (0.0)	0 (0.0)	2 (8.3)	0 (0.0)	0 (0.0)	2 (8.3)
Constipation	0 (0.0)	0 (0.0)	0 (0.0)	0 (0.0)	3 (14.3)	0 (0.0)	2 (9.5)	1 (4.8)	1 (4.2)	0 (0.0)	1 (4.2)	0 (0.0)
Diarrhea	0 (0.0)	0 (0.0)	0 (0.0)	0 (0.0)	1 (4.8)	0 (0.0)	0 (0.0)	1 (4.2)	1 (4.2)	0 (0.0)	1 (4.2)	0 (0.0)
Appetite increase	1 (5.6)	1 (5.6)	0 (0.0)	0 (0.0)	5 (23.8)	0 (0.0)	3 (14.3)	2 (9.5)	3 (12.5)	1 (4.2)	1 (4.2)	1 (4.2)
Appetite decrease	0 (0.0)	0 (0.0)	0 (0.0)	0 (0.0)	0 (0.0)	0 (0.0)	0 (0.0)	0 (0.0)	0 (0.0)	0 (0.0)	0 (0.0)	0 (0.0)
Nausea, vomiting	0 (0.0)	0 (0.0)	0 (0.0)	0 (0.0)	0 (0.0)	0 (0.0)	0 (0.0)	0 (0.0)	0 (0.0)	0 (0.0)	0 (0.0)	0 (0.0)
Urination problems	0 (0.0)	0 (0.0)	0 (0.0)	0 (0.0)	1 (4.8)	0 (0.0)	1 (4.8)	0 (0.0)	1 (4.2)	1 (4.2)	0 (0.0)	0 (0.0)
Sexual problems	0 (0.0)	0 (0.0)	0 (0.0)	0 (0.0)	1 (4.8)	1 (4.8)	0 (0.0)	0 (0.0)	0 (0.0)	0 (0.0)	0 (0.0)	0 (0.0)
Palpitations	0 (0.0)	0 (0.0)	0 (0.0)	0 (0.0)	1 (4.8)	0 (0.0)	1 (4.8)	0 (0.0)	0 (0.0)	0 (0.0)	0 (0.0)	0 (0.0)
Orthostatic hypotension	2 (11.1)	1 (5.6)	1 (5.6)	0 (0.0)	1 (4.8)	0 (0.0)	1 (4.8)	0 (0.0)	1 (4.2)	1 (4.2)	0 (0.0)	0 (0.0)
Vertigo	0 (0.0)	0 (0.0)	0 (0.0)	0 (0.0)	1 (4.8)	1 (4.8)	0 (0.0)	0 (0.0)	0 (0.0)	0 (0.0)	0 (0.0)	0 (0.0)
Sweating	0 (0.0)	0 (0.0)	0 (0.0)	0 (0.0)	1 (4.8)	0 (0.0)	1 (4.8)	0 (0.0)	2 (8.3)	0 (0.0)	2 (8.3)	0 (0.0)
Increased temperature	1 (5.6)	1 (5.6)	0 (0.0)	0 (0.0)	0 (0.0)	0 (0.0)	0 (0.0)	0 (0.0)	1 (4.2)	0 (0.0)	1 (4.2)	0 (0.0)
Tremor	0 (0.0)	0 (0.0)	0 (0.0)	0 (0.0)	0 (0.0)	0 (0.0)	0 (0.0)	0 (0.0)	0 (0.0)	0 (0.0)	0 (0.0)	0 (0.0)
Disorientation	0 (0.0)	0 (0.0)	0 (0.0)	0 (0.0)	0 (0.0)	0 (0.0)	0 (0.0)	0 (0.0)	0 (0.0)	0 (0.0)	0 (0.0)	0 (0.0)
Yawning	1 (5.6)	0 (0.0)	1 (5.6)	0 (0.0)	1 (4.8)	0 (0.0)	1 (4.8)	0 (0.0)	2 (8.3)	0 (0.0)	2 (8.3)	0 (0.0)
Weight gain	1 (5.6)	1 (5.6)	0 (0.0)	0 (0.0)	8 (38.1)	3 (14.3)	4 (19.0)	1 (4.8)	3 (12.5)	1 (4.2)	1 (4.2)	1 (4.2)

ASEC: Antidepressant Side-Effect Checklist(43, 44), Adapted for self-administration in insomnia disorder patients. The items 'difficulty sleeping' and 'drowsiness' in the ASEC-21 were replaced by the items 'difficulty waking up', 'drowsiness in the morning', 'drowsiness in the afternoon', 'restless sleep' and 'vivid dreams'.
This questionnaire was sent to those participants who were on treatment at week 12 or as soon as possible after discontinuation after week 6 and before week 12. 68 participants were on treatment after week 6; missings on this questionnaire were 4, 1, 0 in the treatment groups respectively.

Table S10. Treatment satisfaction among participants who started treatment and in the per protocol group per treatment group. Values are numbers of participants No(total%)(valid%), unless stated otherwise

Outcome measure	Placebo	Mirtazapine	Amitriptyline
Treatment satisfaction	N=25	N=27	N=26
Overall, how satisfied are you with the treatment with tablets from the DREAMING trial?			
Very satisfied	1 (4.0) (5.0)	6 (22.2) (24.0)	4 (15.4)(16.7)
Satisfied	8 (32.0) (40.0)	7 (25.9) (28.0)	7 (26.9) (29.2)
Neither satisfied nor dissatisfied	6 (24.0) (30.0)	5 (18.5) (20.0)	9 (34.6) (37.5)
Dissatisfied	3 (12.0) (15.0)	3 (11.1) (12.0)	3 (11.5)(12.5)
Very dissatisfied	2 (8.0) (10.0)	4 (14.8) (16.0)	1 (3.8) (4.2)
Missing	5 (20.0)	2 (7.4)	2 (7.7)
Time to first improvement	N=25	N=27	N=26
When did you first notice an improvement in your sleep after starting the tablets?			
I have not noticed any improvement	11 (44.0) (55.0)	4 (14.8)(16.0)	5 (19.2)(20.8)
Right on the first night	1 (4.0) (5.0)	9 (33.3) (36.0)	4 (15.4) (16.7)
After some nights	5 (20.0) (25.0)	7 (25.9) (28.0)	9 (34.6)(37.5)
After some weeks	2 (8.0) (10.0)	4 (14.8)(16.0)	4 (15.4) (16.7)
After some months	1 (4.0) (5.0)	1 (3.7) (4.0)	2 (7.7) (8.3)
Missing	5 (20.0)	2 (7.4)	2 (7.7)
Evaluation of treatment duration	N=15	N=15	N=20
What do you think of the treatment duration (16 weeks)?			
This was too short for me	5 (33.3) (33.3)	5 (33.3) (33.3)	6 (30.0) (31.6)
This was (about) right for me	8 (53.3) (53.3)	8 (53.3) (53.3)	11 (55.0) (57.9)
This was too long for me	2 (13.3) (13.3)	2 (13.3) (13.2)	2 (10.0) (10.5)
Missing	0 (0.0)	0 (0.0)	1 (5.0)

Treatment satisfaction questions were evaluated at week 20 or in case of treatment discontinuation in combination with the next planned questionnaire.

**Table S11.** Sleep treatments other than the trial medication among participants who started treatment and in the per protocol group per treatment group. Values are No (total%)(valid%) for categorical outcomes, and No(%), number of participants for dichotomous outcomes.			
Outcome measure	Placebo	Mirtazapine	Amitriptyline
Additional sleep treatments in the intended treatment period (16 weeks)	N=25	N=27	N=26
Sleep medication prescription (EMR data)			
Yes	5 (20.0) (20.0)*	5 (18.5) (18.5)*	3 (11.5) (11.5)*
Type of sleep medication (multiple answers possible):			
BZRA	3	3	2
Melatonin	1	0	0
Mirtazapine**	1	2	1
Amitriptyline	1	1	0
Other (antihistaminic, antipsychotic)	1	1	0
Intake sleep medication (self-reportage)			
No	15 (60.0)(78.9)	22 (81.5)(88.0)	20 (76.9)(83.3)
Yes	4 (16.0)(21.1)	3 (11.1)(12.0)	4 (15.4)(16.7)
Missing	6 (24.0)	2 (7.4)	2 (7.7)
Type of sleep medication (multiple answers possible):			
BZRA	3	2	4
Melatonin	1	0	0
Mirtazapine	0	1	0
Amitriptyline	0	1	0
Course or non-pharmacological therapy (any)(self-reportage)			
Yes	3 (12.0)(15.8)	3 (11.1)(12.0)	4 (15.4)(16.7)
No	16 (64.0)(84.2)	22 (81.5)(88.0)	20 (76.9)(83.3)
Missing	6 (24.0)	2 (7.4)	2 (7.7)
Additional sleep treatments in the 8 months after treatment in per protocol group	N=15	N=15	N=20
Sleep medication prescription (EMR data)			
Yes	7 (46.7)(46.7)	6 (40.0)(40.0)	12 (60.0)(60.0)
Specify (multiple answers possible):			
BZRA	3	1	4
Melatonin	0	1	0
Mirtazapine	4	6	7
Amitriptyline	1	2	4
Other (antihistaminic, antipsychotic)	0	0	0
Amitriptyline and/or mirtazapine	5	6	9
Intake sleep medication (self-reportage)			
No	6 (40.0)(54.5)	9 (60.0)(64.3)	7 (35.0)(36.8)
Yes	5 (33.3)(45.5)	5 (33.3)(35.7)	12 (60.0)(63.2)
Missing	4 (26.7)	1 (6.7)	1 (5.0)
Specify (multiple answers possible):			
BZRA	2	1	5
Melatonin	0	1	1
Mirtazapine	3	4	5
Amitriptyline	1	1	2
Amitriptyline/mirtazapine (not specified)	0	1	1
Amitriptyline and/or mirtazapine	4	5	8
Non-pharmacological therapy (any)(self-reportage)			
Yes	3 (20.0)(27.3)	2 (13.3)(14.3)	3 (15.0)(15.8)
No	8 (53.3)(72.7)	12 (80.0)(85.7)	16 (80.0)(84.2)
Missing	4 (26.7)	1 (6.7)	1 (5.0)

EMR: electronic medical records in general practice.

** 3 participants in each group received one or more prescription for additional sleep medication in the period they reported to be on treatment (period determined per patient); this was 11.1, 11.5, 12.0% in the mirtazapine, amitriptyline and placebo group respectively.*

***2 participants received a prescription for mirtazapine in the period they reported to be on treatment (i.e. 9 days before the planned end of treatment).*

Table S12. Domains of impairment generated from content analysis of GSII* across entire sample†. Presented as number according to rank importance (1-3) and % of total generated areas of sleep-related quality of life impairment (per rank and total)

	All ranks combined	Rank 1	Rank 2	Rank 3
Emotional regulation	53 (24.7)	17 (22.4)	19 (26.8)	17 (25.0)
Energy/motivation	37 (17.2)	18 (23.7)	8 (11.3)	11 (16.2)
Performance at work/school/daily activities	37 (17.2)	13 (17.1)	17 (23.9)	7 (10.3)
Social functioning	27 (12.6)	7 (9.2)	3 (4.2)	17 (25.0)
Cognitive functioning	20 (9.3)	7 (9.2)	9 (12.7)	4 (5.9)
Frustration/concern/preoccupation with sleep loss	11 (5.1)	3 (3.9)	4 (5.6)	4 (5.9)
Health/well-being‡	9 (4.2)	3 (3.9)	4 (5.6)	2 (2.9)
Outlook‡	9 (4.2)	2 (2.6)	3 (4.2)	4 (5.9)
Relationships/family functioning	7 (3.3)	4 (5.3)	1 (1.4)	2 (2.9)
Daytime sleepiness	3 (1.4)	1 (1.3)	2 (2.8)	0 (0.0)
Appearance	2 (0.9)	1 (1.3)	1 (1.4)	0 (0.0)
Total generated areas	215	76	71	68

*GSII: Glasgow Sleep Impact Index (42), *In your own words: what are the 3 most important consequences of your sleeping problem for your daily life? Which of these are you most concerned about? Put them in order by a number 1 (most), 2 and 3 (least).*
†2 participants did fill out at least one area in which they experienced impairment at baseline but not how they were bothered by this, so these are missing from the quantitative analyse.
‡Physical symptoms were included in Health/well-being, concerns about the future and future health were included in Outlook.

Table S13. Treatment adherence among participants who started treatment per treatment group. Values are numbers of participants No(total%)(valid%), unless stated otherwise.

Outcome measure	Placebo	Mirtazapine	Amitriptyline
	N=25	N=27	N=26
Dosage at follow up prescription			
Single dose	7 (28.0) (31.8)	17 (63.0) (73.9)	14 (53.8) (58.3)
Double dose*	15 (60.0) (68.2)	6 (22.2) (26.1)	10 (38.5) (41.7)
No follow up prescription	3 (12.0)	4 (14.8)	2 (7.7)
Self-reported administration time (minutes before bedtime) Mean (SD)	46.0 (29.1), 20	46.3 (41.2), 24	48.4 (32.5), 24
Blinding			
Do you have any idea which tablets you took?			
No, I have no idea	8 (32.0)(42.1)	7 (25.9) (28.0)	10 (38.5) (43.4)
Yes, I think amitriptyline or mirtazapine	6 (24.0) (31.6)	17 (63.0) (68.0)	11 (42.3) (47.8)
Yes, I think placebo (tablet with no active ingredients)	5 (20.0)(26.3)	1 (3.7) (4.0)	2 (7.7) (8.7)
Missing	6 (24.0)	2 (7.4)	3 (11.5)
Adherence			
Did it happen that you took more tablets per day than you had agreed with your GP?			
No	15 (60.0)(78.9)	22 (81.5) (88.0)	22 (84.6) (91.7)
Yes, on 1 to 10 days	4 (16.0) (21.1)	3 (11.1) (12.0)	2 (7.7) (8.3)
Yes, on more than 10 days	0 (0.0) (0.0)	0 (0.0) (0.0)	0 (0.0) (0.0)
Missing	6 (24.0)	2 (7.4)	2 (7.7)
Did it happen that you forgot to take the tablets?			
No	14 (56.0)(70.0)	18 (66.7)(72.0)	16 (61.5)(66.7)
Yes, on 1 to 10 days	5 (20.0)(25.0)	6 (22.2)(24.0)	6 (23.1)(25.0)
Yes, on more than 10 days	1 (4.0)(5.0)	1 (3.7)(4.0)	2 (7.7)(8.3)
Missing	5 (20.0)	2 (7.4)	2 (7.7)
*Did it happen that you decided not to take the tablets?***			
No	19 (76.0)(95.0)	12 (44.4)(50.0)	19 (73.1)(79.2)
Yes, on 1 to 10 days	1 (4.0)(5.0)	6 (22.2)(25.0)	2 (7.7)(8.3)
Yes, on more than 10 days	0 (0.0)(0.0)	6 (22.2)(25.0)	3 (11.5)(12.5)
Missing	5 (20.0)	3 (11.1)	2 (7.7)
Pill count			
PDC >80%	20 (80.0)(90.9)	25 (92.6)(92.6)	23 (88.5)(92.0)
Missing	3 (12.0)	0 (0.0)	1 (3.8)
Pill count			
PDC>90%	17 (68.0)(77.3)	23 (85.2)(85.2)	20 (76.9)(80.0)
Missing	3 (12.0)	0 (0.0)	1 (3.8)

Dosage at follow up prescription: Registered dosage at follow up prescription. Blinding/Adherence: self-reported, assessed at week 20 or in case of treatment discontinuation in combination with the next planned questionnaire. Pill count: adherence by pill count (individually calculated percentage of days covered based on pill count of tablets returned). PDC: percentage of days covered, i.e. number of tablets provided minus the number of tablets returned to the investigator or reported as lost divided by the number of days on the study (correcting for dose) multiplied by 100, cut-offs >80% and >90% were reported..

**Three participants (one in each group) reported in the evaluation questionnaire that after they had received a follow-up prescription for double dose, they had returned to structural single dose*

*** Reported reasons for deliberately missed tablets were improved sleep, side-effects, driving, or insufficiently effective.*
